# Multi-convolutional neural networks for cotton disease detection using synergistic deep learning paradigm

**DOI:** 10.1371/journal.pone.0324293

**Published:** 2025-05-27

**Authors:** Afira Aslam, Syed Muhammad Usman, Muhammad Zubair, Amanullah Yasin, Muhammad Owais, Irfan Hussain

**Affiliations:** 1 Department of Creative Technologies, Faculty of Computing and Artificial Intelligence, Air University, Islamabad, Pakistan; 2 Department of Computer Science, Bahria School of Engineering and Applied Sciences, Bahria University, Islamabad, Pakistan; 3 Interdisciplinary Research Center for Finance and Digital Economy, King Fahd University of Petroleum and Minerals, Dhahran, Saudi Arabia; 4 Department of Mechanical and Nuclear Engineering, Khalifa University, Abu Dhabi, United Arab Emirates; Graphic Era Deemed to be University, INDIA

## Abstract

Cotton is a major cash crop, and increasing its production is extremely important worldwide, especially in agriculture-led economies. The crop is susceptible to various diseases, leading to decreased yields. In recent years, advancements in deep learning methods have enabled researchers to develop automated methods for detecting diseases in cotton crops. Such automation not only assists farmers in mitigating the effects of the disease but also conserves resources in terms of labor and fertilizer costs. However, accurate classification of multiple diseases simultaneously in cotton remains challenging due to multiple factors, including class imbalance, variation in disease symptoms, and the need for real-time detection, as most existing datasets are acquired under controlled conditions. This research proposes a novel method for addressing these challenges and accurately classifying seven classes, including six diseases and a healthy class. We address the class imbalance issue through synthetic data generation using conventional methods like scaling, rotating, transforming, shearing, and zooming and propose a customized StyleGAN for synthetic data generation. After preprocessing, we combine features extracted from MobileNet and VGG16 to create a comprehensive feature vector, passed to three classifiers: Long Short Term Memory Units, Support Vector Machines, and Random Forest. We propose a StackNet-based ensemble classifier that takes the output probabilities of these three classifiers and predicts the class label among six diseases—Bacterial blight, Curl virus, Fusarium wilt, Alternaria, Cercospora, Greymildew—and a healthy class. We trained and tested our method on publicly available datasets, achieving an average accuracy of 97%. Our robust method outperforms state-of-the-art techniques to identify the six diseases and the healthy class.

## Introduction

Agriculture is the primary industry for growing plants, harvesting crops, and raising livestock to produce extra food for the world’s population. Crops, dairy products, edibles, and other main agricultural products are essential to daily living [1]. A little over 40% of the world’s population work in agriculture. Fewer people are employed in agriculture now than in the past [2]. Cotton yield in some of the major countries is shown in Table 1.

**Table 1:** Cotton production in major countries.

Major countries	Production, in thousand metric tons
China	5879
India	5334
United States	3815
Brazil	2678
Pakistan	1306
Australia	1197
Turkey	827
Uzbekistan	577
Argentina	327
Mali	311

Artificial intelligence (AI) is a type of automation used in agriculture to increase productivity, handle difficulties, and find solutions to issues in diverse crop fields [3]. AI can predict crop yield by incorporating technology to collect data on leaf diseases, soil moisture, meteorological conditions, pest infestations, and crop growth. With AI, employing robots, drones, and sensors in agriculture, agronomists can generate high-quality images of crops, increasing the detection output. AI’s main applications in farming are its versatility, performance, accuracy, and cost-effectiveness [4].

**Fig 1:**
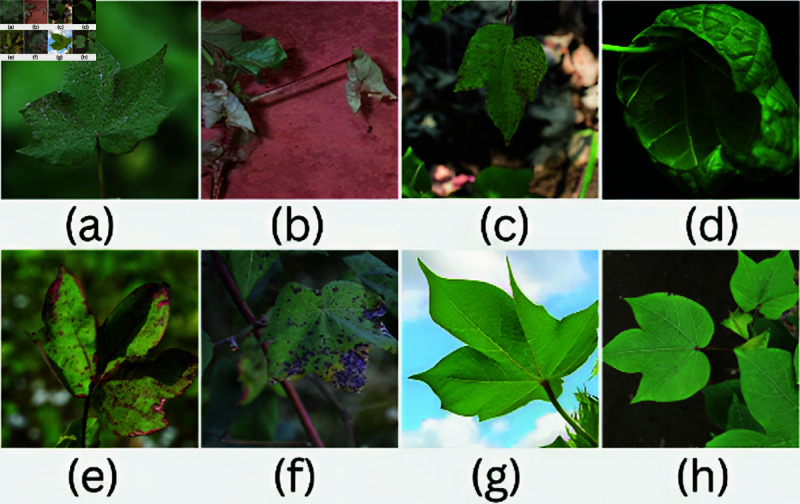
Representative sample images from each category used in the multi-class classification task. (a) Grey mildew, (b) Fusarium wilt, (c) Cercospora, (d) Curl virus, (e) Bacterial blight, (f) Alteranaria, (g–h) Healthy.

The agriculture datasets have been broken down into smaller pieces, and their trends and behaviors have been analyzed for handling a large volume of data [5]. AI should be able to handle agriculture datasets using machine learning and deep learning [28] domains that improve the machines and increase prediction accuracy. Crop disease is a crucial problem in agriculture since it significantly damages crops [6]. The biggest threat to agriculture is crop disease, which reduces yields and the quality and amount of food produced. Cotton is an important economic crop that aids in creating natural fiber. It promotes the expansion of the textile sector. Fig 1 shows seven diseases in cotton plants that are commonly found. These diseases affect the yield of the cotton crop, which causes heavy losses to the farmers.

AI offers a way to determine plant diseases precisely. Deep learning is an automated approach that helps precisely and accurately recognize agricultural diseases while saving money and time. Early and precise recognition of damaged cotton plant leaves is essential in computer vision because of the disease and the changing environment. The complexity is increased by the image’s angle, background, and noise in it. The visual symptoms help in the identification of the disease. Contrarily, it is difficult to create an image with a complex background [7]. The damaging biological risks, such as diseases and pests during the cotton-growing crop times, cost agronomists a tremendous amount of money. Deep learning systems can classify and forecast the damaged cotton crop. Images of the cotton crop are subjected to image processing for segmentation, classification, and identification to provide agricultural scientists with high-quality data. The deep learning algorithms use the segmented image to distinguish between healthy and diseased leaves.

### Key contribution

A robust deep learning system is essential for addressing the practical challenges encountered in real-world agricultural settings. These challenges include diverse symptoms of diseases, such as varying sizes and colors of lesions, which can complicate accurate diagnosis. Additionally, occlusion, where leaves are partially covered by other leaves or objects, poses a significant obstacle to clear image capture and analysis. Variations in leaf appearance, including differences in shapes, sizes, and textures, further add to the complexity. To tackle these issues, we proposed a comprehensive system to manage and overcome these challenges effectively, ensuring reliable and accurate performance in diverse agricultural environments. Contributions of this research to the body of knowledge include:

- Developed a novel method for data augmentation by combining conventional methods and custom Style GANs to reduce class imbalance problems.
- Introduced feature-level fusion scheme by combining features extracted from MobileNet and VGG16 to create a comprehensive feature vector
- Proposed an ensemble classifier using StackNet by integrating Long Short Term Memory Units (LSTM), Support Vector Machine (SVM), and Random Forest to classify cotton crop diseases accurately.
- Achieved an average accuracy of 97% on publicly available datasets, surpassing state-of-the-art techniques in classifying seven classes, including six diseases and a healthy class.

The rest of the paper is organized as follows: the section Literature review provides the related work. The proposed methodology is presented in the section Proposed Methodology and contains a dataset description, whereas results are presented by varying experimental steps in the section Result. A detailed discussion comparing the state of the art is presented in the section Discussion, and the final section Conclusion concludes this research and presents future directions.

## Literature review

A typical method for automated disease detection involves four steps. First is data acquisition, which can be performed using a camera mounted in the field, drones, or a mobile device. After data acquisition, the next step is to preprocess the data to remove the noise and mitigate the class imbalance problem. In the third step, a feature vector is extracted that distinguishes between multiple classes, followed by the classification, which is the last step. Researchers have recently proposed multiple methods for automated disease detection on cotton crops using machine and deep learning models. Table 2 provides a critical analysis of the state-of-the-art methods proposed in recent years for classifying diseases on cotton lead. Deep learning was used by Singh *et al*. [9] and Devi *et al*. [10] to improve plant disease classification. Researchers [9, 11] used the ResNet152V2 model, accomplishing an astounding 97% categorization accuracy. However, Devi *et al*. used ELM to explore the SSADN-PLDDC technique and achieved an impressive 97.87% accuracy. Few researchers [10] neglected a more thorough examination of how hyperparameters affected the effectiveness of the ELM. Combining the two strategies and utilizing the strength of ResNet-based designs with meticulous hyperparameter optimization to maximize the potential of the ELM presents an exciting opportunity.

**Table 2:** Comparison of state-of-the-art methods proposed by researchers in recent years for automated detection of cotton diseases.

Title	Year	Dataset	Method	Accuracy	Limitations
Nazeer *et al*. [46]	2024	Kaggle Dataset and proprietary collection of 1349 images	CNN	99%	Binary classification
Parashar *et al*. [47]	2024	Real-time dataset from drones	MobileNetV2, CNN-based model	99.91%	Focus on diseases caused by pathogens only
Wenjuan *et al*. [48]	2024	Self-built cotton leaf disease dataset	Improved SSD (Single Shot MultiBox Detector) combined with MobileNetV2	3.8% increase	Binary dataset, low performance metric
Rai *et al*. [49]	2023	Self-built cotton leaf disease dataset	CNN	97.98%	Classification on 4 types
Shrivastava *et al*. [50]	2023	Self-built cotton leaf disease dataset	CNN	99.67%	Spread of disease is not discussed
Singh *et al*. [9]	2023	Plant Village Dataset	ResNet152V2	97%	Segmentation of disease is not covered
Mohanavel *et al*. [12]	2022	Very high-resolution images from 450km above	Modified Classifier Logic for Crop Monitoring	85%	Combine mapping, machine-to-machine (M2M), and cloud computing technologies
Devi *et al*. [10]	2022	Dataset on plant diseases from Kaggle repository	SSADN-PLDDC technique with extreme learning machine (ELM)	97.87%	Limited dataset, Lack of analysis on the effect of hyperparameters
Noon *et al*. [16]	2021	Cotton leaf disease publicly available	EfficientNet and MobileNet model	99.95%	Limited data, Lack of analysis on the effect of preprocessing techniques, and only 4 classes were considered
Zekiwos *et al*. [33]	2021	Primary dataset is self-collected, and secondary dataset is obtained from MelakaWorker agricultural research center	CNN	96.40%	Effectiveness of various hyperparameters on the suggested deep learning model’s performance not examined, did not compare with state-of-the-art models
Patil *et al*. [2]	2021	Every image was gathered via a daily survey using an IoT-based system camera and sensor installed in a crop field	CNN	98%	The proposed methodology for the detection of diseases in the cotton plant is time-consuming
Ye *et al*. [34]	2020	120 samples collected from Guangxi site and Hainan site	Binary logistic regression	80%	Examining the variations in spectral response characteristics in Fusarium wilt and other yellowing phenomena
Pechucho *et al*. [35]	2020	ImageNet dataset	DNN inception-V3 pre-trained model	90%	Multiple crops can be detected using the same methods; this study can be extended to find fair use of pesticides after disease detection
Shakeel *et al*. [36]	2020	Public dataset on Cercospora Leaf Spot	K mean clustering and SVM	96%	Size of dataset can be increased; work is only focused on a single disease

Mohanavel *et al*. [12] used high-altitude photos taken from an astounding 450 km above the planet’s surface to monitor crops. With a changed classifier logic, they achieved an accuracy of 85%. This method represents a paradigm shift from traditional ground-based evaluations and shows the promise of remote sensing in disease identification. This technology was expanded by Wang *et al*. [13] by using unmanned aerial vehicles to obtain high-resolution photos. Their accuracy rate with the KMSEG classifier was 88.39%. The synergy between these investigations emphasizes how remote sensing has the potential to revolutionize disease detection. Nonetheless, a comprehensive assessment methodology that includes preprocessing methods, a range of evaluation measures, and implications for practical applicability would benefit both investigations.

With the help of the Deep Neural Networks Inception-V3 pre-trained model and the ImageNet dataset, Pechucho *et al*. [14] obtained an impressive 90% accuracy. Their innovative proposal to expand their system to identify other crops demonstrates the possibility of a single strategy in various agricultural contexts. On the other hand, Shakeel *et al*. [15] targeted study on Cercospora Leaf Spot detection showed that *k*-mean clustering and SVM could detect the disease with 96% accuracy. Combining these findings could spur the development of an all-encompassing strategy for managing multi-crop diseases. However, both studies could strengthen their findings by working with larger datasets and refining their methodologies to cover a broader range of diseases. Noon *et al*. [16] and Xavier *et al*. [17] offer two different strategies for tackling the challenges of incomplete data and identifying multiple diseases. Noon *et al*.’s [16] work, using EfficientNet and MobileNet models, demonstrates an impressive 99.95% accuracy in diagnosing cotton leaf diseases, showcasing the effectiveness of cutting-edge models in situations with limited data.

Researchers [18, 20] have applied multiple methods for the detection and classification of plant diseases, including techniques like the *k*-means algorithm and neural networks, which show good results in the automated detection of diseases on cotton leaves with accuracies between 85% and 89.56%. Moreover, using RGB and HSV components with Artificial Neural Networks also shows an efficient way of disease detection [68]. Preprocessing methods transform color images into grayscale images and also help in disease detection using textural details. Color space segmentation, modified feature maps, Support Vector Machine classification [70], and Gabor filters [71] have also been utilized in literature to detect disease accurately. Dimensionality reduction and scatter matrices are employed to extract key features from images of cotton leaves. ANN [21], along with various image processing techniques, have been used to achieve high accuracy of cotton disease detection. Rothe and Kshirsagar [22] emphasize taking pictures using digital cameras and then post-processing them with Low Pass and Gaussian filters. Seven Hu’s moments are used to generate the features for the classifier training, which are then further segmented using an active contour model. Utilizing this feature vector for disease classification allows the feed-forward back propagation method to be applied.

The image processing technique is also used [23–27, 29] to provide an automated method for disease diagnosis on cotton leaves. The SVM classifier, which has an accuracy of 98.47%, is used for classification using extracted features such as texture and color. Filtering, background removal, and enhancement are preprocessing operations. Color-based segmentation is used to extract sick segments from cotton leaves. Notably, [30] explores a novel approach by focusing on specific lesions and patches rather than complete leaves. This expands the dataset without increasing image count, facilitating multi-disease identification on the same leaf. However, manual symptom separation remains necessary, limiting complete automation. Despite this, the method exhibits enhanced accuracy, showcasing the potential of deep learning techniques for plant disease diagnosis when sufficient data is available, even if not fully exhaustive in representing all practical scenarios. The potential of three-band multispectral images to identify ramularia leaf blight is evaluated across altitudes [31]. While infection levels correlated with altitude increase, distinguishing between illness severity levels posed challenges. Despite varying accuracy, the study underscores the promise of low-cost multispectral devices for detecting cotton ramularia blight. Similarly, another study [32] focuses on automated early-stage cotton illness identification through image processing. Employing the *k*-means clustering technique for segmentation, the hybrid approach integrates texture and color feature extraction.

SVM classified Cercospora cotton leaves with an accuracy of 96%. Exploring the role of mobile robots in agriculture [41], a robot designed for crop inspection is constructed, modeled, and controlled similarly to differential-drive vehicles. Successful autonomous navigation is ensured via odometry and cameras, proving effective for electro-mechanical row crop navigation. Addressing precise self-localization challenges in agriculture, Liu’s report advocates using an above-ground image coupled with crop and weed semantics. The technology demonstrates the potential for precision agriculture applications. For disease identification, Zekiwos and Bruck [33] utilizes GLCM, thresholding, segmentation, and feature extraction. Digital images of RGB leaf captures are processed, considering color transformation and extraction of texture features. In [34], convolutional neural networks and image processing are harnessed, achieving 96% accuracy for classifying cotton leaf diseases. Researchers [35] focus on disease detection, operating parallel processes for healthy and defective leaf images, employing image manipulation techniques, underscoring the importance of robust training in disease diagnosis.

We have identified multiple research gaps by performing the literature review of cotton leaf disease detection. Firstly, data processing predominantly occurred in controlled environments, neglecting real-world variability. Secondly, limited dataset sizes hindered training deep learning models with robust generalization capabilities. Thirdly, prevalent class imbalances posed challenges to model performance. Fourthly, most deep learning research in disease detection focused on binary or small-scale multi-class classification, neglecting broader disease scenarios. Lastly, exploring disease spread on leaf images to estimate affected areas remains largely unexplored.

Our study addresses the above-highlighted research gaps by introducing a novel approach, which not only contributes to filling the existing void in the literature but also paves the way for future investigations. We developed a novel deep ensemble framework tailored to improve the accuracy of cotton disease classification, successfully addressing the issues of class imbalance and symptom variability. Our approach features a creative data augmentation scheme, which employs a customized StyleGAN and traditional methods such as scaling, rotating, transforming, shearing, and zooming to produce a well-balanced dataset. Additionally, our framework includes a dual-stage fusion process that merges feature sets from MobileNet and VGG16 into a comprehensive feature vector. This vector is then processed by a StackNet-based ensemble classifier combining Long and LSTM, SVM, and RF, achieving an outstanding average accuracy of 97% across two publicly available datasets. This performance significantly exceeds current methods in identifying and classifying six distinct diseases and a healthy condition.

## Proposed methodology

**Fig 2:**
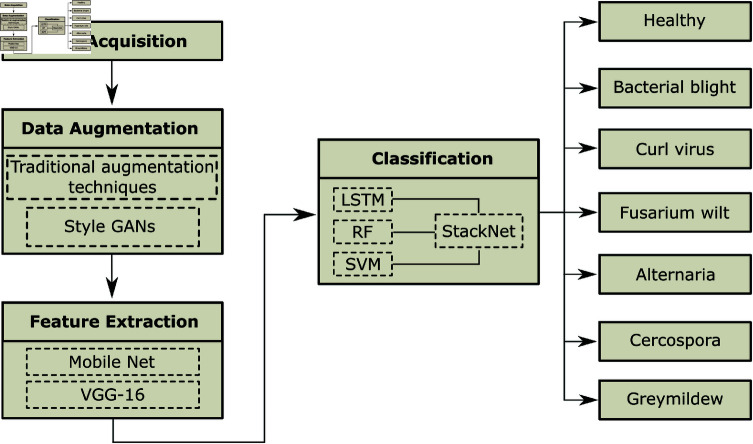
Flowchart of the proposed model for automated detection of cotton crop disease.

Different machine learning and deep learning models under different experimental settings have been tested to develop an accurate and robust method for disease diagnosis. Fig 2 shows the proposed methodology. It consists of four steps: data acquisition, preprocessing, feature extraction, and classification. The public dataset has been used in this study, and to overcome the problem of data scarcity and imbalance, conventional augmentation techniques, including scale, rotate, shear transform, and zooming, along with StyleGAN, which is a state-of-the-art deep learning model for data augmentation, has been customized and used for data augmentation. In the third step, we propose a multi-convolutional neural network fusion-based feature extraction method that concatenates features obtained from VGG16 and MobileNetV2 by removing the fully connected layers of both architectures.

In the last step, we propose a StackNet meta-learning ensemble model that uses SVM, LSTM, and Random Forest as base models for accurately classifying seven classes, including six diseases and one healthy image class. The use of LSTM, despite its typical application for sequential data, is beneficial as it captures temporal dependencies or patterns within the feature vectors that traditional classifiers like SVM and Random Forest might overlook. By integrating these diverse methods, the strength of LSTM in handling sequential patterns is leveraged alongside the robustness of Random Forest in managing non-linear relationships and the effectiveness of SVM in high-dimensional spaces, thereby enhancing overall model performance. Table 3 presents the proposed architecture of StyleGAN, which is implemented to generate synthetic data to reduce the issue of class imbalance. The use of synthetic data only during training, and highlighting that model evaluation was carried out on real, diverse, and unaltered samples. This helps validate the model’s real-world applicability while controlling the effects of any biases introduced by synthetic data.

**Table 3:** Architecture of proposed StyleGAN for data augmentation.

Component	Output resolution	Number of parameters
Generator		
Mapping network	–	2,097,152
Constant input	4 × 4	–
Synthesis network		
4 × 4 to × 8	8 × 8	4,194,304
8 × 8 to 16 × 16	16 × 16	2,097,152
16 × 16 to 32 × 32	32 × 32	1,048,576
32 × 32 to 64 × 64	64 × 64	524,288
64 × 64 to 128 × 128	128 × 128	262,144
128 × 128 to 224 × 224	224 × 224	
Discriminator		
224 × 224 to 128 × 128	128 × 128	
128 × 128 to 64 × 64	64 × 64	524,288
64 × 64 to 32 × 32	32 × 32	1,048,576
32 × 32 to 16 × 16	16 × 16	2,097,152
16 × 16 to 8 × 8	8 × 8	4,194,304
8 × 8 to 4 × 4	4 × 4	8,388,608
Fully connected layer	1	4,096

StyleGAN leverages a series of advanced techniques for generating highly realistic images. The process begins with the transformation of an input latent code *z*, sampled from a normal distribution, into an intermediate latent space *W* through a mapping network, described by W=f(z), where *f* represents the mapping function implemented via fully connected layers. StyleGANs address the problem of data scarcity by generating high-quality synthetic images that are indistinguishable from real images. This increases the dataset size and ensures diversity and variety in the augmented data, thus enhancing the model’s ability to generalize better. A key feature, Adaptive Instance Normalization (AdaIN), is applied as


$$\text{AdaIN}(x,y)=y_{s}\frac{x-\mu (x)}{\sigma (x)}+y_{b},$$

adjusting the style of the generated image, where *y*_*s*_ and *y*_*b*_ are scale and bias parameters from the style vector *y*, and μ(x) and σ(x) are the mean and standard deviation of the feature map *x*.

Progressive growing allows the model to start with low-resolution images, adding layers to increase resolution and facilitating a coarse-to-fine learning approach. The truncation trick, modulating the latent space *W* with a parameter ψ, is used to adjust the balance between diversity and fidelity, yielding $W^{\prime }=\psi W\;+\;(1\;-\;\psi )\overset{―}{W}$, with $\overset{―}{W}$ as the average style vector.

To introduce stochastic variations, noise *n* is added to feature maps *x*, resulting in $x^{\prime }=x+n\cdot \alpha$, where α is a per-feature learned scaling factor.

The GAN loss function, optimizing the generator *G* and discriminator *D*, is formulated as ${ℒ}_{GAN}(G,D)={𝔼}_{z\sim p_{z}}[\log(1-D(G(z)))]+{𝔼}_{x\sim p_{data}}[\log D(x)]$, where *z* and *x* represent latent vectors and real images, respectively.

The VGG16 architecture, renowned for its simplicity and depth, significantly influenced convolutional neural network designs. Central to VGG16 is its uniform use of 3×3 convolutional layers stacked sequentially to capture complex patterns within images. The convolution operation mathematically represents this design choice:

$$f_{\text{out}}=\text{ReLU}(W*f_{\text{in}}+b)$$ (1)

where $f_{\text{in}}$ and $f_{\text{out}}$ are the input and output feature maps, *W* represents the weights of the 3×3 convolutional filters, *b* is the bias, and ReLU denotes the Rectified Linear Unit activation function.

Following each convolutional block, VGG16 employs max pooling to reduce spatial dimensions, thereby condensing information and reducing computation for subsequent layers:

$$f_{\text{reduced}}=\text{MaxPool}(f_{\text{out}})$$ (2)

The network culminates in fully connected layers, designed to flatten the high-level features extracted by the convolutional layers into a vector, which is then mapped to the desired number of classes for classification tasks. Table 4 presents the architecture and list of parameters of the VGG16 used in this research for feature extraction.

**Table 4:** VGG16 Architecture with detailed list of parameters for automated feature extraction.

Layer Type	Output Size	Parameters
Input	224×224×3	0
Conv3x3 + ReLU (x2)	224×224×64	38,720
MaxPooling	112×112×64	0
Conv3x3 + ReLU (x2)	112×112×128	221,440
MaxPooling	56×56×128	0
Conv3x3 + ReLU (x3)	56×56×256	1,475,328
MaxPooling	28×28×256	0
Conv3x3 + ReLU (x3)	28×28×512	5,899,776
MaxPooling	14×14×512	0
Conv3x3 + ReLU (x3)	14×14×512	7,079,424
MaxPooling	7×7×512	0
Fully Connected + ReLU	4096	102,764,544
Fully Connected + ReLU	4096	16,781,312
Fully Connected + Softmax	1000	4,097,000

MobileNetV2 introduces the concept of depth-wise separable convolutions and a residual structure. Due to this, it provides high accuracy with less computational cost. Depth-wise separable convolution consists of depthwise convolution followed by point-wise convolution.

$$f_{\text{depth-wise}}=\text{depth-wiseConv}(f_{\text{in}})$$ (3)

$$f_{\text{pointwise}}=\text{Conv1x1}(f_{\text{depth-wise}})$$ (4)

It applies a single filter per channel, and pointwise convolution (1×1 convolution) combines the output of the depth-wise convolution across multiple channels. This operation enables the use of fewer parameters.

The inverted residual block of MobileNetV2 consists of an initial expansion layer (1×1 convolution), a depth-wise convolution. Then, a projection layer (1×1 convolution) is used to condense the feature maps, interspersed with shortcut connections. This design optimizes information flow and efficiency:

$$f_{\text{expanded}}=\text{Conv1x1}(f_{\text{in}},\text{expansion factor})$$ (5)

$$f_{\text{projected}}=\text{Conv1x1}(\text{ReLU6}(\text{depth-wiseConv}(f_{\text{expanded}})))$$ (6)

**Fig 3:**
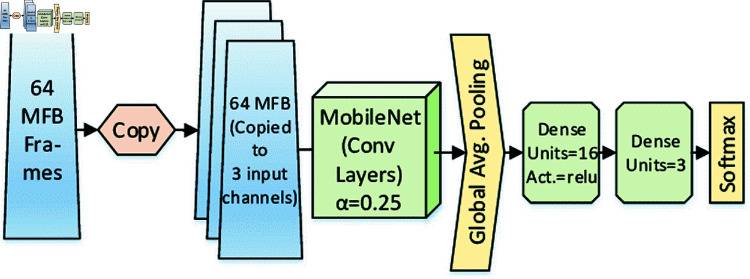
Architecture of the MobileNet proposed for feature extraction.

Table 5 details the trainable parameters required in MobileNet V2. MobileNetV2 is a lightweight architecture that classifies images in real-time mobile devices with low computational complexity. Fig 3 shows the layer-wise diagram of the MobileNetV1. MobileNetV2 builds on the original MobileNetV1 using depth-wise separable convolutions to reduce computational cost and achieve high accuracy. Depth-wise convolution and point-wise convolution are the main reasons behind the low computational cost of MobileNetV2.

**Table 5:** MobileNetV2 architecture with detailed list of parameters for automated features extraction.

Layer type	Output size	Parameters
Input	224×224×3	0
Conv3x3 + ReLU6	112×112×32	864
Bottleneck (x1)	112×112×16	5,136
Bottleneck (x2)	56×56×24	28,224
Bottleneck (x3)	28×28×32	47,840
Bottleneck (x4)	14×14×64	88,064
Bottleneck (x3)	14×14×96	137,088
Bottleneck (x3)	7×7×160	320,160
Bottleneck (x1)	7×7×320	472,064
Conv1x1 + ReLU6	7×7×1280	409,600
Average Pooling	1×1×1280	0
Fully Connected + Softmax	1000	1,280,000

$$Y=F(X)+X_{1}$$ (7)

where *Y* denotes the output, *F*(*X*) the convolutional process, and *X* the input feature map. We have combined features extracted from VGG16 and MobileNetV2 to form a single feature vector, which is then fed as input to the three different classifiers, including LSTM, RF, and SVM. The output of these classifiers is combined using StackNet to get the final output. The ensemble classification method improves the accuracy of disease detection. The process of ensemble classification using Stacknet is as follows:

$$F=\text{MetaClassifier}\left(\sum_{i=1}^{n}w_{i}\cdot O_{i}\right)$$ (8)

In the above-mentioned equation, *F* is the output produced by the StackNet, which is the final classification result. The ensemble classifier effectively integrates these inputs to produce the final decision and improves overall classification accuracy.

**Fig 4:**
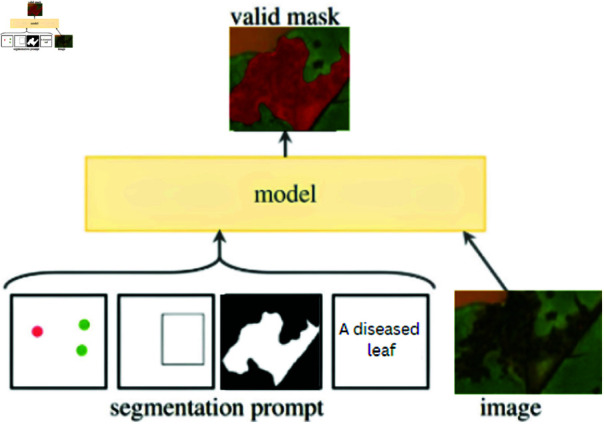
Segment Anything Model (SAM) framework for segmentation of cotton crop leaves.

Meta has launched a new "Segment Anything" project to create two crucial parts. The Segment Anything paradigm (SAM) [43] is a foundation paradigm for quick picture segmentation. This draws inspiration from the field of NLP, where big datasets (worth billions of tokens) and foundation models are becoming standard. The project results in the simultaneous development of a sizable dataset, a segmentation model, and a data engine. The scientists picked picture segmentation as the starting point for these enormous models and datasets since it is one of the fundamental computer vision problems. Image segmentation offers a variety of possible applications in both science and artificial intelligence. Fig 4 contains segment anything models general framework.

The proposed methodology employs pre-trained deep-learning models to distinguish between healthy and diseased cotton leaves and identify the disease type from leaf images. Machine learning classifiers are utilized to extract the features of VGG16 and MobileNetV2. The classifiers are being trained to categorize images into disease groups, with performance metrics being used to compare and identify the optimal classifier. A “Segment Anything Model” is used for precise pixel-level segmentation of diseased areas, aiding in calculating disease-covered area percentage.

We have trained the model by changing hyperparameters, including different numbers of epochs, optimizers, activation functions, number of neurons in a dense layer, and ensemble classification. MobileNetV2 performs well and achieved test accuracies between 0.92 and 0.97, whereas, DenseNet121 achieved 0.89 test accuracy. Although results from other models vary, this research points out that MobileNetV2 and DenseNet121 show promise as powerful options for identifying and mapping out diseases on cotton leaves. After performing an ablation study, the proposed method was finalized. The proposed method is compared with the state-of-the-art existing methods for cotton disease detection, and the proposed method performs well in terms of both the number of classes and accuracy. The proposed method achieves a testing accuracy of 0.97 with seven classes, including six diseases and a healthy class.

Jupyter BBox is used to annotate the images for the SAM model for pixel-level segmentation to get the area affected by the disease. First, we bring in the required libraries and then create a BBoxWidget. This lets users outline the areas they’re interested in with bounding boxes. Next, we load the mask predictor and feed the image into it, using the MaskPredictor’s predict() method. This process gives us the expected masks, scores, and logits. A BoxAnnotator and a MaskAnnotator are generated to annotate identified objects with bounding boxes and segmentation masks. A detection object is formed from the predicted masks, and the object with the most significant area is selected.

**Fig 5:**
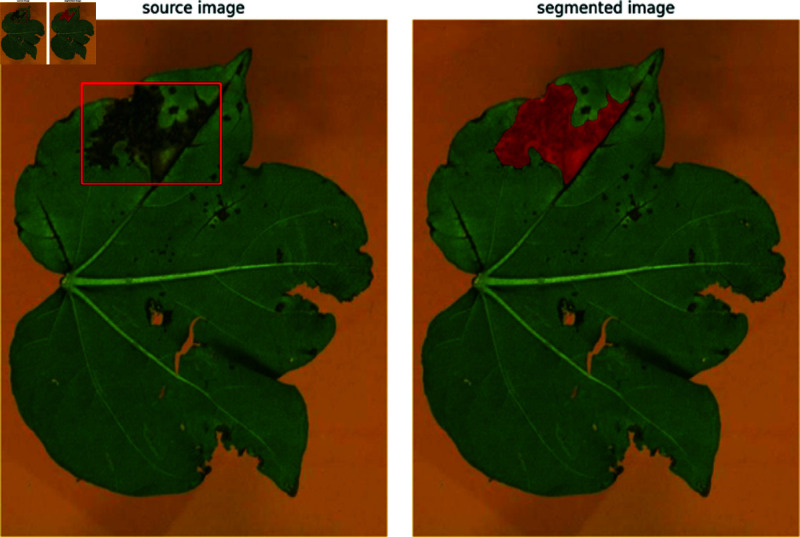
Segmentation results obtained from SAM on cotton leaf image.

**Fig 6:**
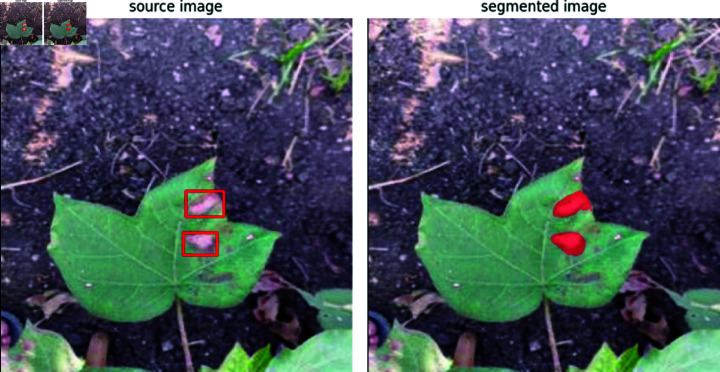
Results of multiple segmentation on single leaf image using SAM.

The source and segmented images are then created using the BoxAnnotator and MaskAnnotator’s methods. SAM effectively segments various disease-affected leaf images from controlled environments or field conditions. Using an annotated dataset, SAM can aid disease classification from leaf images with reduced resource and time requirements. This underscores SAM’s potential for practical disease classification applications. Fig 5 shows the source and segmented images, and Fig 6 shows multiple bounding boxes.

For feature extraction, a machine learning pipeline extracts features from images using a pre-trained VGG16 and MobileNetV2, trains different machine learning models on the collected features, and evaluates the classifier’s performance on a test set. The first section of the code imports necessary libraries, including Keras, which is used to load the VGG16 and MobileNetV2 models. Next, the VGG16 model is loaded with pre-trained weights from the ImageNet dataset. In subsequent steps, this model is utilized to extract features from images.

A function named “extract_features” processes images, applying Keras utilities, VGG16, and MobileNetV2 models. Features and labels are flattened, stored in arrays, and saved as numpy arrays. These are used for training and testing sets. Logged through Scikit-learn’s Pipeline and GridSearchCV, machine learning models are evaluated using hyperparameter tuning with a parameter grid. Grid search cross-validation assesses performance, and accuracy is measured using Scikit-learn’s score function. StackNet—a powerful ensemble learning model, is used later to classify diseases. StackNet excels in combining diverse base models, enhancing predictive performance by leveraging their unique strengths. The combined predictions are calculated by linearly combining base models along with their weights, as shown in equation 2.

$$P\_ensemble(X)=w_{1}.Pm_{1}(X)+w_{2}.Pm_{2}(X)+....+w_{k}.Pm_{k}(X),$$ (9)

where P_ensemble(X) represents the meta-model prediction and *m*_1_, ... *m*_*k*_ represents base models. The loss function is

$$\sum_{i=1}^{N}loss(Y_{i},P\_ensemble(x_{i})),$$ (10)

## Results

### Dataset and experimental setup

This research has used two publicly available datasets [44, 45]. The combined dataset used in this study includes seven classes, out of which six classes are diseases and one is healthy. The diseases represented in the dataset cover a diverse range of symptoms and appearances, providing a comprehensive basis for evaluating the performance of our classification models. Class imbalance and limited data availability against many classes are challenges. To address the class imbalance issue, we first performed conventional data augmentation techniques such as rotation, scaling, flipping, and cropping. We also employed StyleGAN, a state-of-the-art generative adversarial network, to generate synthetic images. Combining these synthetic images with the real images helped reduce the class imbalance problem. Table 6 provides several images in each class. We split the images into 80,10 and 10 ratios for train, validation, and testing.

**Table 6:** Information of dataset with number of images in each class for training, validation, and testing.

Image class	Training	Validation	Testing
Greymildew	832	104	104
Fusarium wilt	776	97	97
Cercospora	784	98	98
Curl virus	776	97	97
Bacterial blight	744	93	93
Alternaria	776	97	97
Healthy	792	99	99

### Ablation study

Table 7 presents the accuracy achieved using DT, *k*NN, LSTM, RF, SVM, and an Ensemble Classifier (StackNet) with feature vector extracted from VGG16 and MobileNetV2. Decision Tree classifier shows low performance with MobileNetV2 features (62% to 56%), while KNN demonstrates a significant improvement (65% to 87%). The LSTM classifier performs well with both feature sets, showing an improvement from 84% to 87%, and the Random Forest classifier also improves accuracy from 80% to 83%. The SVM classifier maintains high accuracy, improving slightly from 86% to 88%. The Ensemble Classifier (StackNet) outperforms all individual classifiers by a significant margin, achieving a consistently high accuracy of 97% with both feature sets. The percentage gain of the Ensemble Classifier over the best individual classifier (SVM) is approximately 12.79% for VGG16 features and 10.23% for MobileNetV2 features, highlighting the robustness and effectiveness of the proposed method in integrating multiple model predictions.

**Table 7:** Accuracy of multiple classifiers with a deep-learning-based feature set

Classifier	Accuracy (VGG_16 features	Accuracy (MobileNetV2 features)
Decision Tree (DT)	0.62	0.56
*k* Nearest Neighbor (KNN)	0.65	0.87
Long Short-Term Memory Units (LSTM)	0.84	0.87
Random Forest (RF)	0.8	0.83
Support Vector Machine (SVM)	0.86	0.88
Ensemble Classifier (StackNet)	0.97	

**Fig 7:**
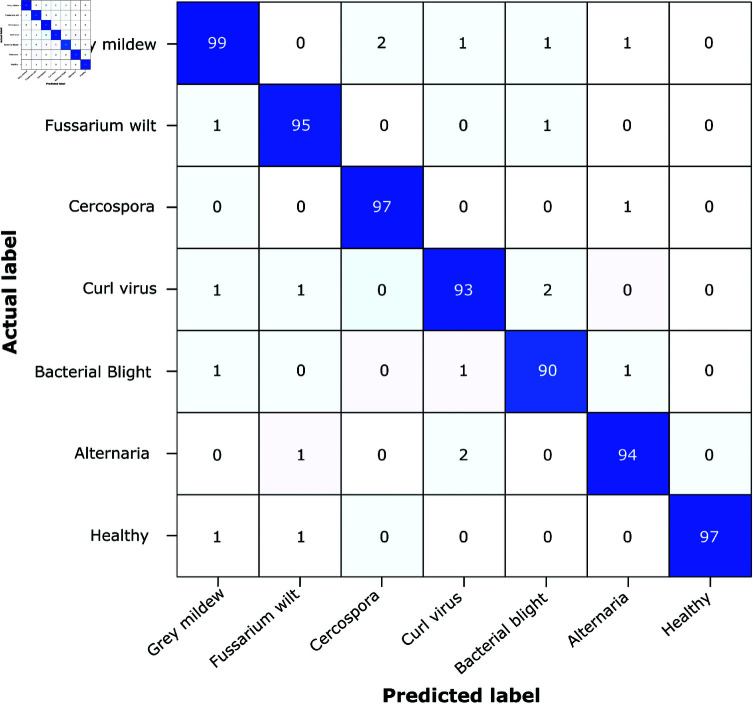
Confusion matrix for the proposed ensemble classifier.

Fig 7 shows the confusion matrix of the proposed ensemble classifier (StackNet), demonstrating its superior performance and reliability. StackNet combines the output of different classifiers that results in achieving high accuracy with low false positive rate. Confusion matrix shows that the ensemble classification performs better.

**Fig 8:**
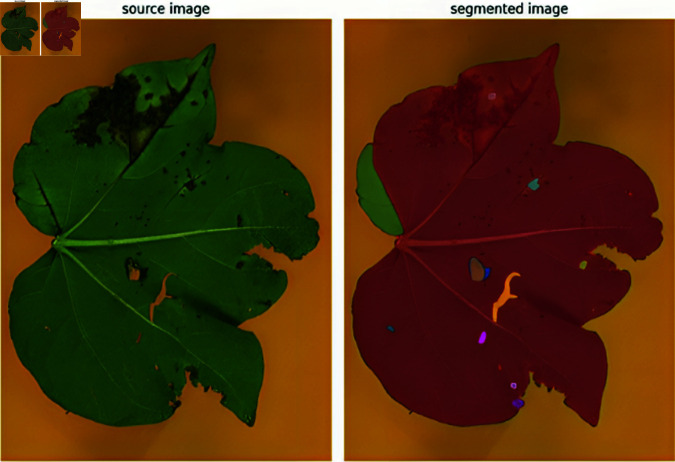
The whole leaf image segmented using SAM.

The disease portion can be extracted by removing the healthy portion of the image, which is around 17%. Images are segmented into two regions, i.e., healthy and diseased, to compute the area under disease. It is done by pixel-level segmentation and counting the total number of pixels that are affected by the disease. This helps in estimating the severity of the disease. Fig 8 shows the leaf image segmented using the SAM. It identifies the diseased portion of the leaf, which is then highlighted to estimate the disease-affected area.

### Comparison with state of the art methods

The results are derived from different datasets, including Plant Village [59] and Roboflow [60], with either binary or multiclass classification. Among the listed models, DenseNet-121 achieves the highest accuracy (0.99) on the Plant Village dataset [59], followed by MobileNetV2 (0.91), also trained on the same dataset. The proposed ensemble model (MobileNetV2 + VGG16 + StackNet) achieves the third-highest accuracy (0.97), but it is the only approach that integrates multiple datasets (Plant Village + Roboflow), demonstrating its robustness in handling a more diverse dataset and a 7-class classification problem. Other notable models include MobileNetV2 with additional dense layers (0.98) and Xception (0.94), utilizing the Plant Village [59] dataset for multiclass classification. While the proposed method has a higher parameter count (143.4M) compared to lightweight models such as MobileNetV2 (3.5M) and DenseNet-121 (8.0M), its ability to generalize across multiple datasets justifies its complexity. Studies utilizing the Roboflow dataset [60] have reported an accuracy of 0.96 using a position attention-based capsule network [73] for multi-class classification, and 0.93 using a YOLO-based model [75]. In general, the table highlights that most existing methods rely on a single dataset, whereas the proposed ensemble model effectively uses multiple datasets, making it a more robust and scalable solution for real-world cotton disease detection.

Evaluating a model on a combined dataset, as in our study, introduces a broader range of data variability, including differences in image quality, lighting conditions, background noise, and disease severity. This diversity makes the classification task more complex and better simulates real-world scenarios where such variations are common. In contrast, many prior studies were conducted using a single, often curated dataset like PlantVillage, which may not fully capture these variations and can lead to overly optimistic performance metrics. By training and testing our model on a combination of the PlantVillage and Roboflow datasets, we ensured that it was exposed to a more heterogeneous set of samples. This approach serves as a more rigorous evaluation of the model’s robustness, generalization ability, and adaptability to diverse input conditions. Notably, the Roboflow dataset is widely recognized for its greater complexity due to less uniform image characteristics, making it a more challenging benchmark. Our model’s superior performance on this dataset, compared to recent state-of-the-art methods, underscores its practical utility and reliability in real-world applications.

### External dataset testing

To further substantiate the robustness and generalizability of our proposed model, a publicly available cotton leaf disease dataset [76] is used as a benchmark for external testing and independent evaluation. This dataset provides a rich, real-world representation of disease variability and environmental diversity, making it ideal for validating deep learning-based classification models in agricultural domains. The dataset comprises a total of 2137 original images along with 7000 augmented images, designed to enhance training efficacy for deep learning models. These images span seven classes, representing both healthy and diseased cotton leaves across different conditions, including Bacterial blight (250 images), Cotton curl virus (431 images), Herbicide growth damage (280 images), Leaf hopper Jassids (225 images), Leaf reddening (578 images), Leaf variegation (116 images), and Healthy leaf (257 images). The dataset is available at https://data.mendeley.com/datasets/b3jy2p6k8w/2

Each image captures critical disease-specific features such as leaf discoloration, curling, wilting, necrosis, and other symptomatic indicators. The dataset is particularly valuable as it includes images collected from real field environments during different growth stages of the cotton plant. These were taken under varying lighting conditions, natural backgrounds, and imaging devices, reflecting the type of noise and variability encountered in practical settings. The image resolutions vary, including high-resolution formats such as 3000×4000 pixels, 2239×2239 pixels, and 1597×1597 pixels, which contributes to a heterogeneous and challenging dataset for model evaluation. All images were captured through systematic field surveys conducted under expert supervision, ensuring both the accuracy and representativeness of disease labeling. This dataset not only serves as a critical resource for external validation but also acts as a robust benchmark to assess a model’s performance beyond controlled training environments. By evaluating our model on this external dataset, we can verify its ability to generalize well across unseen and more complex data distributions, thereby supporting its practical applicability in precision agriculture.

**Table 8:** Comprehensive comparative analysis of the proposed model versus existing methods for cotton disease detection and other applications in the agricultural domain. Results are based on different PlantVillage [59] and Roboflow [60] datasets with multiclass classification.

Author	Year	Model	#Par (M)	Accuracy	Dataset	Classification
Wang *et al*. [54]	2023	MobileNetV2	3.5	0.91	Plant village [59]	Multiclass
Wang *et al*. [54]	2023	MobileNetV2 + more dense layers	4	0.98	Plant village [59]	Multiclass
Alam *et al*. [53]	2024	Xception	22.9	0.94	Plant village [59]	Multiclass
Alam *et al*. [53]	2024	Resnet152	60.4	0.93	Plant village [59]	Multiclass
Alam *et al*. [53]	2024	EfcientNetB4	19.3	0.96	Plant village [59]	Multiclass
Eunice *et al*. [55]	2022	DenseNet-121	8.0	0.99	Plant village [59]	Multiclass
Bhatheja *et al*. [57]	2021	Deep CNN	5.3	0.90	Plant village [59]	Multiclass
Bhujade *et al*. [73]	2025	Position attention-based capsule network	6.2	0.96	Roboflow [60]	Multiclass
Maqbool *et al*. [75]	2023	YOLO-based Object Detection Model	7.2	0.93	Roboflow [60]	Multiclass
Proposed Method	-	Ensemble (MobileNetV2+VGG16eak+StackNet)	143.4	0.97	Plant village [59]+Roboflow [60]	Multiclass (7 classes)

Table 9 presents a comparative analysis of our proposed model’s performance against several recent state-of-the-art methods on the publicly available cotton leaf disease dataset [76]. Although the accuracy of our model (0.964) is marginally lower than some existing models that achieved up to 0.992, it is important to emphasize that our model was evaluated entirely on unseen data from this dataset. In contrast, the referenced models often utilized subsets of this same dataset for both training and validation, leading to more favorable but potentially biased accuracy outcomes.

**Table 9:** Performance comparison of the proposed model on the cotton leaf disease dataset [76] against State-of-the-Art methods for robustness evaluation.

Author	Year	Model	Accuracy
Bishshash *et al*. [76]	2024	Inception V3	0.960
Nirob *et al*. [77]	2025	Atrous Spatial Pyramid Pooling with Squeeze-and-Excitation blocks	0.992
Patra *et al*. [78]	2024	Modified MobileNet	0.984
Rahman *et al*. [79]	2025	DenseNet169 with Convolutional Block Attention Module (CBAM)	0.962
		EfficientNetB1 with CBAM	0.992
Proposed Method	-	Ensemble (MobileNetV2+VGG16+StackNet)	0.964

The objective of this external validation was not solely to outperform existing methods on this dataset, but rather to test the generalizability and robustness of our model when applied to entirely new data under different conditions. Our model had no prior exposure to this dataset during training, which makes the achieved performance particularly noteworthy. Additionally, our model has already demonstrated strong and consistent results on two diverse datasets, including “PlantVillage” [59] and Roboflow [60], both of which encompass variations in lighting, background, disease severity, and image quality.

The combination of high performance on varied training datasets and consistent accuracy on this unseen external dataset illustrates the robustness and adaptability of our approach. This validates the reliability of the proposed model beyond ideal or curated data environments. Hence, these results strongly support the practical applicability of our model in real-time settings, where data is often heterogeneous and unpredictable. Our model’s ability to generalize well, even on unseen data, confirms its potential for real-time cotton disease classification in practical agricultural scenarios.

### Visual explanation of model decisions

**Fig 9:**
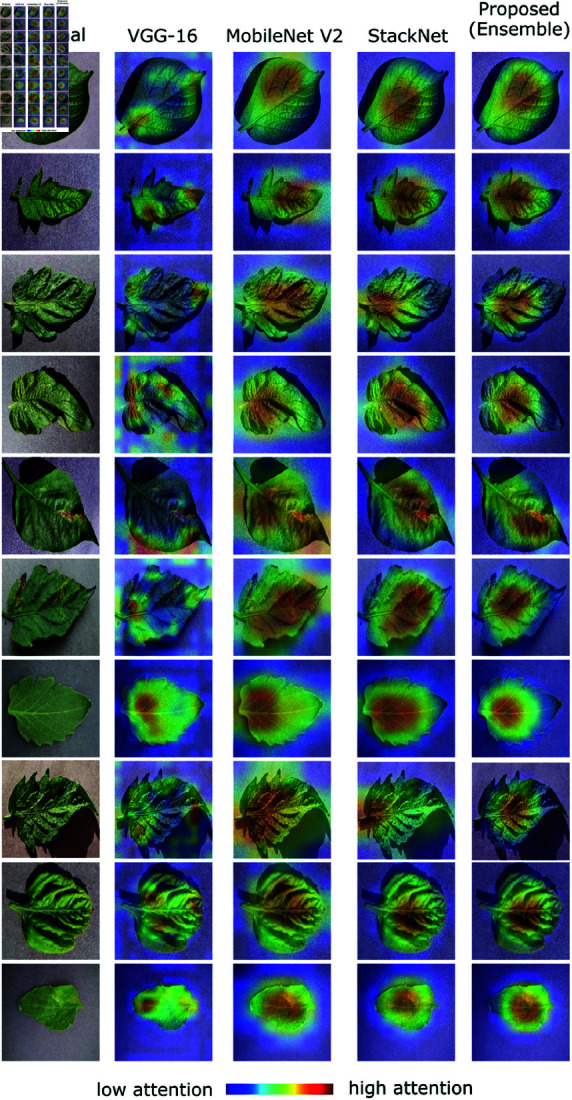
Grad-CAM visualizations illustrating model interpretability for cotton disease classification on samples from Plant Village dataset [59]. The heatmaps highlight the discriminative regions used by each model to make predictions. The images exhibit attention on relevant symptomatic regions.

The interpretability of the deep learning model gain trust among agricultural experts and farmers, so, we incorporated explainable AI techniques, specifically Gradient-weighted Class Activation Mapping (Grad-CAM). Grad-CAM offers visual explanations by highlighting the regions in the input image that the model focuses on while making a classification decision. This provides insights into the inner workings of the model and helps validate whether the decisions are being made for the right reasons. We applied Grad-CAM on correctly classified test samples from the Plant Village [59] dataset and visualized the attention maps generated for VGG-16, MobileNet V2, StackNet, and our proposed ensemble model. As shown in Fig 9, the heatmaps reveal that the proposed ensemble model consistently attends to the most relevant symptomatic regions of cotton leaves, such as areas of necrosis, discoloration, or curling, which are key visual indicators for disease identification.

In comparison, the individual models (VGG-16 and MobileNet V2) sometimes highlight broader or less specific regions, which could lead to misinterpretation or reduced confidence in the model’s focus. The StackNet, while more refined than individual models, also shows scattered attention in some cases. The ensemble model, by aggregating and refining the strengths of these networks, demonstrates a more precise and localized focus on disease-affected areas. These visual explanations serve two crucial purposes: they provide transparency for the decision-making process and offer a layer of validation that the model is not relying on irrelevant features or background artifacts. For domain experts and farmers, this interpretability builds trust and supports adoption, as visual evidence aligns with agronomic knowledge of disease symptoms. By integrating Grad-CAM into our analysis, we not only demonstrate the robustness of our model but also make a significant step toward explainable and trustworthy AI in agricultural diagnostics.

## Discussion

Cotton crop classification is a crucial aspect of precision agriculture, enabling accurate yield estimation, disease detection, and optimal resource allocation. However, researchers faced several challenges that hindered the development of robust cotton crop classification systems. Firstly, data scarcity and imbalance pose a significant hurdle, with limited availability of high-quality data and disproportionate representation of certain crop classes. Secondly, extracting comprehensive features from cotton crop images is challenging due to their complex and variable nature. Hence, achieving accurate classification across seven cotton crop classes remains difficult due to the inherent similarity between some classes.

To further verify the generalization capability of the proposed model and address concerns related to potential overfitting due to synthetic data, we performed external validation using a publicly available cotton leaf disease dataset [76], which was completely unseen during training. Despite no prior exposure to this dataset, the proposed model achieved a high accuracy of 0.964, which is competitive with other recent methods that used this same dataset for both training and testing. This external validation reinforces the model’s robustness and confirms its ability to generalize effectively to real-world data with different image resolutions, disease severities, and environmental backgrounds.

The critical examination of results reveals that the proposed method outperforms several notable methods, including ResNet152V2 [9], Modified Classifier Logic for Crop Monitoring [12], and SSADN-PLDDC technique with ELM [10], all of which were designed for datasets with fewer classes. The proposed method demonstrates superior performance compared to methods utilizing EfficientNet and MobileNet [16], Convolutional Neural Networks [2, 33], Binary logistic regression [1], DNN inception-V3 pre-trained mode [35], K-mean clustering and SVM [36], KMSEG classifier [37], MLR, MLRb, SVM, RFT [38], Transfer learning [69] with GoogleNet CNN [39, 40]. Feature Selection and classification using SVM [41]. Confirming that the best-performing classifier is SVM, with the MobileNetV2 feature set. This in-depth review highlights how well the suggested approach works and its ability to scale and adapt as it deals with an increasing variety of categories. The accurate results have been achieved due to an ensemble learning method that combines StyleGANs, VGG16, and MobileNetV2 within the StackNet framework. This approach takes advantage of automated feature extraction, concatenates the features, passes it to three different classifiers, and performs ensemble classification using StackNet.

Together with strong results on the PlantVillage [59] and Roboflow [60] datasets, the model’s competitive performance on unseen data highlights its potential for deployment in real-time cotton disease classification applications.

### Key findings and potential significance

The proposed ensemble method, trained with categorical cross-entropy loss, RMSProp optimizer, and softmax activation, achieves an accuracy of 97%. We introduce a novel method for disease detection that helps in dealing with real-world challenges such as varying disease symptoms, occlusion from overlapping leaves, and diversity in leaves in terms of shape, size, and texture. A novel data augmentation technique has been developed to address the issue of data scarcity and class imbalance. Feature level fusion of multi-convolutional neural combined with ensemble classification techniques is a novel approach to improve the accuracy of disease detection with an increased number of disease classes. This method combines the strengths of convolutional neural networks for feature extraction and classification. As a result, it accurately identifies six specific diseases and a healthy class in cotton crops. Moreover, the robustness of the model was demonstrated through external testing on a completely unseen, publicly available dataset, where it achieved a high classification accuracy. This result highlights the model’s strong generalization capability, suggesting its potential for effective deployment in real-world scenarios beyond controlled training environments.

### Limitations and future directions

The primary limitation of this study is the possibility of overfitting due to the controlled environment in which the data was collected and the models were trained. This controlled setting may not accurately reflect the variability and complexity of real-world field conditions, leading to lower performance when the models are applied in ream time scenarios. Moreover, a small dataset restricts the system’s effectiveness and its ability to generalize well across diverse scenarios. To partially mitigate this limitation, we performed external validation using a completely unseen, real-world cotton leaf disease dataset. The model achieved a competitive accuracy of 0.964, despite not being exposed to this dataset during the training or validation phase. This indicates a strong generalization capability of the proposed model and supports its robustness against overfitting, even when applied to new field data from different domains.

While the external validation shows encouraging signs of real-world applicability, further improvements are necessary to ensure consistent performance in diverse and uncontrolled environments. Future research must focus on real-time data acquisition and pre-processing to increase the robustness of model in real environments [74]. Improving model interpretability and transparency will ensure AI decisions are understandable to farmers and agricultural professionals. Developing user-friendly interfaces and mobile applications will aid practical field use. Expanding the dataset with diverse images of diseased leaves will improve generalization and accuracy. Advanced algorithms for precise disease coverage localization will optimize pesticide usage, while refined segmentation techniques will better handle overlapping symptoms of multiple diseases on a single leaf.

## Conclusion

We have addressed three problems: data scarcity and imbalance, comprehensive feature set extraction, and accurate classification of seven classes of cotton crops. It has been observed that conventional data augmentation techniques are insufficient to address the problem of imbalance and scarcity. Therefore, we propose a customized StyleGANs network for data augmentation and conventional methods. Similarly, a comprehensive feature set has been obtained by removing the fully connected VGG16 and MobileNet v2 layers and concatenating the features obtained from these two models. For accurate classification, we propose an ensemble classifier based on StackNet with SVM, LSTM, and Random Forest as base classifiers, and the the output is used in meta-learning. The proposed method has been applied to public datasets to validate the accuracy. It outperforms the existing methods of cotton crop disease detection in terms of both accuracy and number of classes. To further demonstrate the generalizability and robustness of the proposed model, we conducted external validation using a completely unseen publicly available dataset. Our methodology show results comparable to other state-of-the-art models, which affirms the ability of the model to handle diverse data in the real world and validates its potential for reliable use in practical agricultural applications.
