# Expression of Concern: Multi-convolutional neural networks for cotton disease detection using synergistic deep learning paradigm

**DOI:** 10.1371/journal.pone.0343102

**Published:** 2026-02-17

**Authors:** The PLOS One Editors

After this article [1] was published, the following concerns were noted:

- The article cited as Reference 58 was retracted before [1] was published.
- The articles cited as Reference 23-27, 29, and 68-71 do not appear to support the corresponding cited statements [1].

In addition, the *PLOS One* Editors have concerns about the article’s compliance with the PLOS Authorship policy.

In light of the cumulative issues, the *PLOS One* Editors issue this Expression of Concern. Readers are advised to interpret the article [1] with caution.

Additionally, the following errors were identified in the References list:

- The article cited as Reference 2 in the published article is incorrectly duplicated as Reference 66.
- The article cited as Reference 3 in the published article is incorrectly duplicated as Reference 6.
- The article cited as Reference 7 in the published article is incorrectly duplicated as Reference 36.
- The article cited as Reference 9 in the published article is incorrectly duplicated as Reference 61.
- The article cited as Reference 10 in the published article is incorrectly duplicated as Reference 63.
- The article cited as Reference 12 in the published article is incorrectly duplicated as Reference 62.
- The article cited as Reference 13 in the published article is incorrectly duplicated as Reference 37.
- The article cited as Reference 14 in the published article is incorrectly duplicated as Reference 35.
- The article cited as Reference 15 in the published article is incorrectly duplicated as Reference 67.
- The article cited as Reference 16 in the published article is incorrectly duplicated as Reference 64.
- The article cited as Reference 17 in the published article is incorrectly duplicated as Reference 38.
- The article cited as Reference 33 in the published article is incorrectly duplicated as Reference 65.
- The article cited as Reference 41 in the published article is incorrectly duplicated as Reference 72.
- The articles cited as References 8, 19, 42, 51, 52, 56, 58, 61-67, and 72 are missing from the text and data of the article.
