# Supplementary material for: Multi-convolutional neural networks for cotton disease detection using synergistic deep learning paradigm
Source: PLoS One. 2025 May 27;20(5):e0324293. doi: 10.1371/journal.pone.0324293 (PMC12112145; doi:10.1371/journal.pone.0324293)
Supplement: Attachment — Submitted filename: 2024-08-17-Response to Editor and Reviewers.pdf [file pone.0324293.s002.pdf]

< Editor >

Dear Editor,

We appreciate your reviewing process of our submitted manuscript titled "Multi-Convolutional Neural Networks for Cotton Disease Detection Using Ensemble Learning" in the PLOS One journal.

We have thoroughly revised our paper according to **"Journal Requirements"** and comments provided by the reviewers. All the revisions are in **red and blue color text** in the revised manuscript. Moreover, a rebuttal file is also provided to address the changes made in the revised paper against individual comments by the reviewers.

Best regards

Dr. Muhammad Owais

< Reviewer 1 >

< Comment >

The authors edited the manuscript according to the comments. Therefore, accepted for the publication.

< Response >

We would like to thank the reviewer and appreciate your reviewing process of our manuscript.

< Reviewer 3 >

< Comment 1 >

The authors have not been able to show the new contribution. This paper does not add anything new to knowledge as the authors dont state the major contributions of the work.

< Response >

Authors would like to thank the reviewer for the valuable comment. We have highlighted the contributions and incorporated them in the Introduction section, **lines 48-57** on **page no. 3**.

Action taken in the revised manuscript

- Developed a novel method for data augmentation by combining conventional methods and custom Style GANs to reduce class imbalance problems.
- Introduced feature-level fusion scheme by combining features extracted from MobileNet and VGG16 to create a comprehensive feature vector
- Proposed an ensemble classifier using StackNet by integrating Long Short Term Memory Units (LSTM), Support Vector Machine (SVM), and Random Forest to classify cotton crop diseases accurately.
- Achieved an average accuracy of 97\% on publicly available datasets, surpassing state-of-the-art techniques in classifying seven classes, including six diseases and a healthy class.

< Comment 2 >

There is no comparative analysis with the state-of-the-art methods.

< Response >

The authors would like to thank the reviewer for the valuable suggestion. We have added a comparison of the proposed method with existing state-of-the-art methods. **Table 8** has been updated in the results section on **page no. 17** and described in the text.

Action taken in the revised manuscript

Table 8. Comprehensive comparative analysis of the proposed model versus existing methods for cotton disease detection and other applications in the agricultural domain

Author	Year	Model	#Par (M)	Accuracy
Sivakumar et al. [53]	2021	InceptionV3	23.9	0.86
Wang et al. [50]	2023	MobileNetV2	3.5	0.88
Wang et al. [50]	2023	MobileNetV2 unfreezing initial layers	3.5	0.88
Alam et al. [51]	2024	Xception	22.9	0.88
Alam et al. [51]	2024	Resnet152	60.4	0.78
Eunice et al. [52]	2022	DenseNet121	8.0	0.89
Wang et al. [50]	2023	MobileNetV2 with more dense layers	3.5	0.90
Bhatheja et al. [54]	2021	Deep CNN	5.3	0.90
Wang et al. [50]	2023	MobileNetV2 with learning scheduler	3.5	0.91
**Proposed Method**	-	Ensemble (MobileNetV2 + VGG16 + StackNet)	143.4	**0.97**

Table 8 presents a comparative analysis of the proposed model versus existing methods for cotton disease detection, focusing on their parameters (#Par in millions) and accuracy. The proposed ensemble model achieves the highest accuracy of 0.97, indicating a 6% performance gain over the next best models. The second-best models [50, 54], with accuracies of 0.91 and 0.90, include MobileNetV2 with a learning scheduler (0.91, 3.5M), MobileNetV2 with more dense layers (0.90, 3.5M), and EfficientNet (0.90, 5.3M). The third-best models [50, 51], with an accuracy of 0.88, include MobileNetV2 (3.5M), MobileNetV2 unfreezing initial layers (3.5M) and Xception (22.9M). Despite the proposed method's high parameter count (143.4M), its 6% performance gain justifies the complexity, significantly outperforming models like MobileNetV2 (3.5M) and DenseNet121 (8.0M), which achieve relatively high accuracy. This highlights the effectiveness of our ensemble model in achieving superior accuracy, balancing parameter size and performance gain, and demonstrating the model's efficacy in cotton disease detection applications.
