# Supplementary material for: Multi-convolutional neural networks for cotton disease detection using synergistic deep learning paradigm
Source: PLoS One. 2025 May 27;20(5):e0324293. doi: 10.1371/journal.pone.0324293 (PMC12112145; doi:10.1371/journal.pone.0324293)
Supplement: Attachment — Submitted filename: Response to Reviewers.pdf [file pone.0324293.s003.pdf]

Dear Editor,

We appreciate your reviewing process of our submitted manuscript titled ***"Multi-Convolutional Neural Networks for Cotton Disease Detection Using Ensemble Learning"*** in the PLOS One journal.

We have revised our paper according to the comments provided by the editor and reviewers. All added text is in Red, with removed text as (~~strike through~~) in the ***"Revised Manuscript with Track Changes".*** Moreover, a ***"Response to Reviewer(s)"*** file is also provided to address the changes made in the revised paper against individual comments by the reviewers. In addition, a clean file ***"Manuscript"*** is also uploaded in separate.

Best regards

## Editor Comments

Comment 1: The study lacks clarity on whether all referenced methods in Table 8 are evaluated using the same dataset. This makes it difficult to accurately compare accuracy metrics across different approaches.

**Response:** We appreciate the reviewer's observation. In Table 8 of the original manuscript, we presented the results of prior studies primarily based on the "PlantVillage" dataset. However, our proposed model was evaluated on a combined dataset comprising both the "PlantVillage" and "Roboflow" datasets. We acknowledge that this discrepancy may have made cross-study comparisons unclear.

To address this, we have revised Table 8 to include recent studies that report performance on the "Roboflow" dataset as well. This ensures a more accurate and meaningful comparison. While two existing studies report slightly higher accuracy on the "PlantVillage" dataset alone, it is important to highlight that our model demonstrates robust performance across both datasets when combined, suggesting better generalization capability. Moreover, we have removed the result of one study which is shows results on a completely different dataset.

Our model's strong performance on the combined datasets "PlantVillage" and "Roboflow" datasets is considered more challenging, further emphasizes its effectiveness and practical applicability.

**Action:** Please see the section "Comparison with State-of-the-Art methods" and Table 8 on page numbers 16-17 in the revised track changes manuscript.

### Comparison with State of the Art methods

The results are derived from different datasets, including Plant Village [57] and Roboflow [58], with either binary or multiclass classification. Among the listed models, DenseNet-121 achieves the highest accuracy (0.99) on the Plant Village dataset [57], followed by MobileNetV2 (0.91), also trained on the same dataset. The proposed ensemble model (MobileNetV2 + VGG16 + StackNet) achieves the third-highest accuracy (0.97), but it is the only approach that integrates multiple datasets (Plant Village + Roboflow), demonstrating its robustness in handling a more diverse dataset and a 7-class classification problem. Other notable models include MobileNetV2 with additional dense layers (0.98) and Xception (0.94), utilizing the Plant Village [57] dataset for multiclass classification. While the proposed method has a higher parameter count (143.4M) compared to lightweight models such as MobileNetV2 (3.5M) and DenseNet-121 (8.0M), its ability to generalize across multiple datasets justifies its complexity. Studies utilizing the Roboflow dataset [58] have reported an accuracy of 0.96 using a position attention-based capsule network [72] for multi-class classification, and 0.93 using a YOLO-based model [73]. In general, the table highlights that most existing methods rely on a single dataset, whereas the proposed ensemble model effectively uses multiple datasets, making it a more robust and scalable solution for real-world cotton disease detection.

Evaluating a model on a combined dataset, as in our study, introduces a broader range of data variability, including differences in image quality, lighting conditions, background noise, and disease severity. This diversity makes the classification task more complex and better simulates real-world scenarios where such variations are common. In contrast, many prior studies were conducted using a single, often curated dataset like PlantVillage, which may not fully capture these variations and can lead to overly optimistic performance metrics. By training and testing our model on a combination of the PlantVillage and Roboflow datasets, we ensured that it was exposed to a more heterogeneous set of samples. This approach serves as a more rigorous evaluation of the model's robustness, generalization ability, and adaptability to diverse input conditions. Notably, the Roboflow dataset is widely recognized for its greater complexity due to less uniform image characteristics, making it a more challenging benchmark. Our model's superior performance on this dataset, compared to recent state-of-the-art methods, underscores its practical utility and reliability in real-world applications.

**Table 8.** Comprehensive comparative analysis of the proposed model versus existing methods for cotton disease detection and other applications in the agricultural domain. Results are based on ~~different~~ PlantVillage [57] and Roboflow [58] datasets with ~~binary or~~ multiclass classification.

| **Author** | **Year** | **Model** | **#Par (M)** | **Accuracy** | **Dataset** | **Classification** |
|---|---|---|---|---|---|---|
| ~~Sivakumar et al.~~ [53] | ~~2021~~ | ~~InceptionV3~~ | ~~23.9~~ | ~~0.96~~ | ~~Akash Zade~~ [56] | ~~Binary~~ |
| Wang et al. [50] | 2023 | MobileNetV2 | 3.5 | 0.91 | Plant village [57] | Multiclass |
| Wang et al. [50] | 2023 | MobileNetV2 + more dense layers | 4 | 0.98 | Plant village [57] | Multiclass |
| Alam et al. [51] | 2024 | Xception | 22.9 | 0.94 | Plant village [57] | Multiclass |
| Alam et al. [51] | 2024 | Resnet152 | 60.4 | 0.93 | Plant village [57] | Multiclass |
| Alam et al. [51] | 2024 | EfcientNetB4 | 19.3 | 0.96 | Plant village [57] | Multiclass |
| Eunice et al. [52] | 2022 | DenseNet-121 | 8.0 | 0.99 | Plant village [57] | Multiclass |
| Bhatheja et al. [54] | 2021 | Deep CNN | 5.3 | 0.90 | Plant village [57] | Multiclass |
| Bhujade et al. [72] | 2025 | Position attention-based capsule network | 6.2 | 0.96 | Roboflow [58] | Multiclass |
| Maqbool et al. [73] | 2023 | YOLO-based Object Detection Model | 7.2 | 0.93 | Roboflow [58] | Multiclass |
| **Proposed Method** | - | Ensemble (MobileNetV2+VGG16+StackNet) | 143.4 | **0.97** | Plant village [57]+Roboflow [58] | Multiclass (7 classes) |

Comment 2: Discrepancies exist in reported accuracy values for references. For example, the manuscript previously stated that Reference 53 had an accuracy of 86%, but the published value was 96.46%. Similarly, Reference 50's accuracy was misrepresented.

**Response:** We appreciate the reviewer for pointing out these discrepancies. Upon review, we found that Reference 53 was based on a different dataset that was not used in our model evaluation. To maintain a fair and consistent comparison, we have removed this reference from Table 8, as now reflected in the revised manuscript. Additionally, the accuracy value for Reference 50 has been corrected accordingly. We have also thoroughly verified all other referenced accuracy values to ensure consistency and accuracy across the manuscript.

**Action:** Please see Table 8 on page numbers 17 in the revised track changes manuscript.

**Table 8.** Comprehensive comparative analysis of the proposed model versus existing methods for cotton disease detection and other applications in the agricultural domain. Results are based on ~~different~~ PlantVillage [57] and Roboflow [58] datasets with ~~binary or~~ multiclass classification.

| Author | Year | Model | #Par (M) | Accuracy | Dataset | Classification |
|---|---|---|---|---|---|---|
| ~~Sivakumar et al.~~ [53] | ~~2021~~ | ~~InceptionV3~~ | ~~23.9~~ | ~~0.96~~ | ~~Akash Zade~~ [56] | ~~Binary~~ |
| Wang et al. [50] | 2023 | MobileNetV2 | 3.5 | 0.91 | Plant village [57] | Multiclass |
| Wang et al. [50] | 2023 | MobileNetV2 + more dense layers | 4 | 0.98 | Plant village [57] | Multiclass |
| Alam et al. [51] | 2024 | Xception | 22.9 | 0.94 | Plant village [57] | Multiclass |
| Alam et al. [51] | 2024 | Resnet152 | 60.4 | 0.93 | Plant village [57] | Multiclass |
| Alam et al. [51] | 2024 | EfcientNetB4 | 19.3 | 0.96 | Plant village [57] | Multiclass |
| Eunice et al. [52] | 2022 | DenseNet-121 | 8.0 | 0.99 | Plant village [57] | Multiclass |
| Bhatheja et al. [54] | 2021 | Deep CNN | 5.3 | 0.90 | Plant village [57] | Multiclass |
| Bhujade et al. [72] | 2025 | Position attention-based capsule network | 6.2 | 0.96 | Roboflow [58] | Multiclass |
| Maqbool et al. [73] | 2023 | YOLO-based Object Detection Model | 7.2 | 0.93 | Roboflow [58] | Multiclass |
| **Proposed Method** | - | Ensemble (MobileNetV2+VGG16+StackNet) | 143.4 | **0.97** | Plant village [57]+Roboflow [58] | Multiclass (7 classes) |

Comment 3: The model is trained in a controlled setting, which may not accurately reflect real-world variability, including lighting conditions, environmental noise, and different imaging devices. This could lead to reduced generalizability when deployed in practical scenarios.

**Response:** We acknowledge the reviewer's concern and have addressed this by evaluating our model on a combined dataset comprising PlantVillage and Roboflow, which differ significantly in terms of image quality, lighting conditions, background complexity, and noise. This combination introduces higher data variability and better reflects real-world scenarios. Unlike single-dataset studies, our approach provides a more rigorous test of generalization. Notably, our model performed strongly on the more challenging Roboflow dataset, demonstrating its robustness and applicability in diverse practical settings.

**Action:** Please see the added text on page number 17 in the revised track changes manuscript.

Evaluating a model on a combined dataset, as in our study, introduces a broader range of data variability, including differences in image quality, lighting conditions, background noise, and disease severity. This diversity makes the classification task more complex and better simulates real-world scenarios where such variations are common. In contrast, many prior studies were conducted using a single, often curated dataset like PlantVillage, which may not fully capture these variations and can lead to overly optimistic performance metrics. By training and testing our model on a combination of the PlantVillage and Roboflow datasets, we ensured that it was exposed to a more heterogeneous set of samples. This approach serves as a more rigorous evaluation of the model's robustness, generalization ability, and adaptability to diverse input conditions. Notably, the Roboflow dataset is widely recognized for its greater complexity due to less uniform image characteristics, making it a more challenging benchmark. Our model's superior performance on this dataset, compared to recent state-of-the-art methods, underscores its practical utility and reliability in real-world applications.

Comment 4: Although the study attempts to address class imbalance through synthetic data generation (StyleGAN and augmentation), reliance on synthetic data might introduce biases that do not accurately represent real-world variations in disease symptoms.

**Response**: We appreciate the reviewer's valuable observation. While synthetic data generation using StyleGAN and augmentation was used to address class imbalance, we recognize the potential risk of introducing biases that may not fully capture the complexity of real-world disease manifestations. To mitigate this, we ensured that the synthetic samples were only used during the training phase and not in validation or testing, which were conducted exclusively on real images from the combined PlantVillage and Roboflow datasets.

**Action:** See 2nd paragraph of the "Proposed Methodology" on page number 8 in the revised track changes manuscript.

In the last step, we propose a StackNet meta-learning ensemble model that uses SVM, LSTM, and Random Forest as base models for accurately classifying seven classes, including six diseases and one healthy image class. The use of LSTM, despite its typical application for sequential data, is beneficial as it captures temporal dependencies or patterns within the feature vectors that traditional classifiers like SVM and Random Forest might overlook. By integrating these diverse methods, the strength of LSTM in handling sequential patterns is leveraged alongside the robustness of Random Forest in managing non-linear relationships and the effectiveness of SVM in high-dimensional spaces, thereby enhancing overall model performance. Table 3 presents the proposed architecture of the StyleGAN, which is implemented to generate synthetic data to reduce the issue of class imbalance. The use of synthetic data only during training, and highlighting that model evaluation was carried out on real, diverse, and unaltered samples. This helps validate the model's real-world applicability while controlling the effects of any biases introduced by synthetic data.

Comment 5: The reported 97% accuracy suggests a potential overfitting issue, especially given that the dataset is relatively small and augmented artificially. More robust validation techniques, such as external dataset testing, would strengthen the claims.

**Response:** We thank the reviewer for raising this important concern. To address the potential overfitting issue, we conducted an external validation using a completely unseen, publicly available cotton leaf disease dataset [74]. Unlike prior studies that used parts of this dataset for both training and testing, our model was evaluated solely on the test set without any prior exposure. Despite this, our model achieved a competitive accuracy of 96.4%, confirming its generalization ability. These results, combined with strong performance on diverse datasets like PlantVillage and Roboflow, demonstrate the robustness and real-world applicability of our model. We have included this external validation and discussion in the revised manuscript (see Section "External Validation Testing" and Table 9). Moreover, the sections “Discussion”, “Key Findings and Potential Significance”, “Limitations and Future Directions”, and “Conclusion” are also updated accordingly.

**Action:** See section “External Dataset Testing” on page number 18, Table 9 on page 19, and “Discussion” section on page 19 and 21, “Key Findings and Potential Significance” section on page 21, “Limitations and Future Directions” on page 21-22, and “Conclusion” section on page 22 in the revised track changes manuscript.

## External Dataset Testing

To further substantiate the robustness and generalizability of our proposed model, a publicly available cotton leaf disease dataset [81] is used as a benchmark for external testing and independent evaluation. This dataset provides a rich, real-world representation of disease variability and environmental diversity, making it ideal for validating deep learning-based classification models in agricultural domains. The dataset comprises a total of 2137 original images along with 7000 augmented images, designed to enhance training efficacy for deep learning models. These images span seven classes, representing both healthy and diseased cotton leaves across different conditions, including Bacterial blight (250 images), Cotton curl virus (431 images), Herbicide growth damage (280 images), Leaf hopper Jassids (225 images), Leaf reddening (578 images), Leaf variegation (116 images), and Healthy leaf (257 images). The dataset is availabe at `https://data.mendeley.com/datasets/b3jy2p6k8w/2`

Each image captures critical disease-specific features such as leaf discoloration, curling, wilting, necrosis, and other symptomatic indicators. The dataset is particularly valuable as it includes images collected from real field environments during different growth stages of the cotton plant. These were taken under varying lighting conditions, natural backgrounds, and imaging devices, reflecting the type of noise and variability encountered in practical settings. The image resolutions vary, including high-resolution formats such as 3000×4000 pixels, 2239×2239 pixels, and 1597×1597 pixels, which contributes to a heterogeneous and challenging dataset for model evaluation. All images were captured through systematic field surveys conducted under expert supervision, ensuring both the accuracy and representativeness of disease labeling. This dataset not only serves as a critical resource for external validation but also acts as a robust benchmark to assess a model's performance beyond controlled training environments. By evaluating our model on this external dataset, we can verify its ability to generalize well across unseen and more complex data distributions, thereby supporting its practical applicability in precision agriculture.

Table 9 presents a comparative analysis of our proposed model's performance against several recent state-of-the-art methods on the publicly available cotton leaf disease dataset [81]. Although the accuracy of our model (0.964) is marginally lower than some existing models that achieved up to 0.992, it is important to emphasize that our model was evaluated entirely on unseen data from this dataset. In contrast, the referenced models often utilized subsets of this same dataset for both training and validation, leading to more favorable but potentially biased accuracy outcomes.

The objective of this external validation was not solely to outperform existing methods on this dataset, but rather to test the generalizability and robustness of our model when applied to entirely new data under different conditions. Our model had no prior exposure to this dataset during training, which makes the achieved performance particularly noteworthy. Additionally, our model has already demonstrated strong and consistent results on two diverse datasets, including "PlantVillage" [61] and Roboflow [62], both of which encompass variations in lighting, background, disease severity, and image quality.

The combination of high performance on varied training datasets and consistent accuracy on this unseen external dataset illustrates the robustness and adaptability of our approach. This validates the reliability of the proposed model beyond ideal or curated data environments. Hence, these results strongly support the practical applicability of our model in real-time settings, where data is often heterogeneous and unpredictable. Our model's ability to generalize well, even on unseen data, confirms its potential for real-time cotton disease classification in practical agricultural scenarios.

**Table 9.** Performance comparison of the proposed model on the cotton leaf disease dataset [81] against State-of-the-Art methods for robustness evaluation

| **Author** | **Year** | **Model** | **Accuracy** |
|---|---|---|---|
| Bishshash et al. [81] | 2024 | Inception V3 | 0.960 |
| Nirob et al. [82] | 2025 | Atrous Spatial Pyramid Pooling with Squeeze-and-Excitation blocks | 0.992 |
| Patra et al. [83] | 2024 | Modified MobileNet | 0.984 |
| Rahman et al. [84] | 2025 | DenseNet169 with Convolutional Block Attention Module (CBAM)<br>EfficientNetB1 with CBAM | 0.962<br>0.992 |
| **Proposed Method** | - | Ensemble (MobileNetV2+VGG16+StackNet) | 0.964 |

## Discussion

To further verify the generalization capability of the proposed model and address concerns related to potential overfitting due to synthetic data, we performed external validation using a publicly available cotton leaf disease dataset [81], which was completely unseen during training. Despite no prior exposure to this dataset, the proposed model achieved a high accuracy of 0.964, which is competitive with other recent methods that used this same dataset for both training and testing. This external validation reinforces the model's robustness and confirms its ability to generalize effectively to real-world data with different image resolutions, disease severities, and environmental backgrounds.

Together with strong results on the PlantVillage [61] and Roboflow [62] datasets, the model's competitive performance on unseen data highlights its potential for deployment in real-time cotton disease classification applications.

## Key Findings and Potential Significance

diseases and a healthy class in cotton crops. Moreover, the robustness of the model was demonstrated through external testing on a completely unseen, publicly available dataset, where it achieved a high classification accuracy. This result highlights the model's strong generalization capability, suggesting its potential for effective deployment in real-world scenarios beyond controlled training environments.

## Limitations and Future Directions

to generalize well across diverse scenarios. To partially mitigate this limitation, we performed external validation using a completely unseen, real-world cotton leaf disease dataset. The model achieved a competitive accuracy of 0.964, despite not being exposed to this dataset during the training or validation phase. This indicates a strong generalization capability of the proposed model and supports its robustness against overfitting, even when applied to new field data from different domains.

While the external validation shows encouraging signs of real-world applicability, further improvements are necessary to ensure consistent performance in diverse and uncontrolled environments. Future research must focus on real-time data acquisition

## Conclusion

and number of classes. To further demonstrate the generalizability and robustness of the proposed model, we conducted external validation using a completely unseen publicly

available dataset. Our methodology show results comparable to other state-of-the-art models, which affirms the ability of the model to handle diverse data in the real world and validates its potential for reliable use in practical agricultural applications.

Comment 6: The study does not provide sufficient interpretability techniques (e.g., Grad-CAM, SHAP) to understand why the model makes certain predictions. Explainable AI methods could enhance trust in the system for agricultural experts and farmers.

**Response:** We appreciate the reviewer's valuable suggestion. In the revised manuscript, we have incorporated the Explainable AI technique Grad-CAM to provide visual explanations for the model's predictions. A new section titled "Visual Explanation of Model Decisions" has been added, along with Figure 9, which presents Grad-CAM visualizations for VGG-16, MobileNet V2, StackNet, and our proposed ensemble model. These visualizations highlight the discriminative regions used by each model, thereby improving transparency and helping end-users understand and trust the model's decision-making process.

**Action:** See section "Visual Explanation of Model Decisions" on page 19 and Figure 9 on page 20 in the revised track changes manuscript.

## Visual Explanation of Model Decisions

The interpretability of the deep learning model gain trust among agricultural experts and farmers, so, we incorporated explainable AI techniques, specifically Gradient-weighted Class Activation Mapping (Grad-CAM). Grad-CAM offers visual explanations by highlighting the regions in the input image that the model focuses on while making a classification decision. This provides insights into the inner workings of the model and helps validate whether the decisions are being made for the right reasons. We applied Grad-CAM on correctly classified test samples from the Plant Village [61] dataset and visualized the attention maps generated for VGG-16, MobileNet V2, StackNet, and our proposed ensemble model. As shown in Figure 9, the heatmaps reveal that the proposed ensemble model consistently attends to the most relevant symptomatic regions of cotton leaves, such as areas of necrosis, discoloration, or curling, which are key visual indicators for disease identification.

In comparison, the individual models (VGG-16 and MobileNet V2) sometimes highlight broader or less specific regions, which could lead to misinterpretation or reduced confidence in the model's focus. The StackNet, while more refined than individual models, also shows scattered attention in some cases. The ensemble model, by aggregating and refining the strengths of these networks, demonstrates a more precise and localized focus on disease-affected areas. These visual explanations serve two crucial purposes: they provide transparency for the decision-making process and offer a layer of validation that the model is not relying on irrelevant features or background artifacts. For domain experts and farmers, this interpretability builds trust and supports adoption, as visual evidence aligns with agronomic knowledge of disease symptoms. By integrating Grad-CAM into our analysis, we not only demonstrate the robustness of our model but also make a significant step toward explainable and trustworthy AI in agricultural diagnostics.

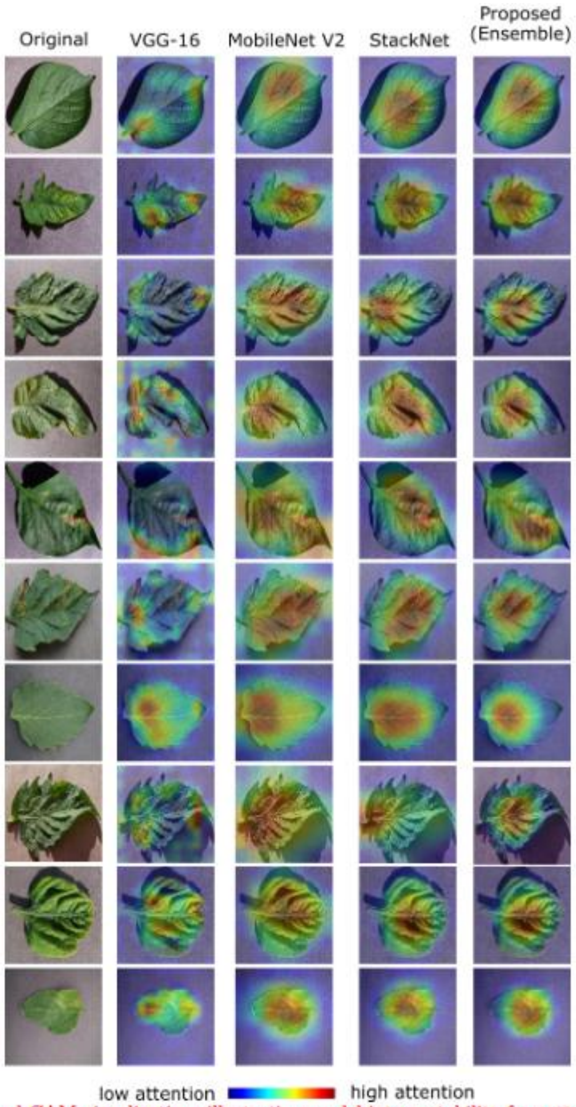


**Fig 9.** Grad-CAM visualizations illustrating model interpretability for cotton disease classification on samples from Plant Village dataset [61]. The heatmaps highlight the discriminative regions used by each model to make predictions. The images exhibit attention on relevant symptomatic regions.
