# Supplementary material for: Multi-convolutional neural networks for cotton disease detection using synergistic deep learning paradigm
Source: PLoS One. 2025 May 27;20(5):e0324293. doi: 10.1371/journal.pone.0324293 (PMC12112145; doi:10.1371/journal.pone.0324293)
Supplement: Attachment — Submitted filename: 2024-06-22-Response to Editor and Reviewers.docx [file pone.0324293.s001.docx]

**< Editor Comments >**

Dear Editor,

We appreciate your review of our submitted manuscript titled “Multi-Convolutional Neural Networks for Cotton Disease Detection Using Ensemble Learning” in the PLOS One journal.

We have thoroughly revised our paper according to the “Journal Requirements” and the comments provided by the “Reviewers”. All the revisions are highlighted in red and blue text in the revised manuscript. We have also included a rebuttal to address the changes made in response to each reviewer's comments.

Best Regards,

Muhammad Owais (Ph.D.)

Corresponding author

[muhammad.owais@ku.ac.ae](mailto:muhammad.owais@ku.ac.ae)

Contanct#: +971-58-135-8344

**< Journal Requirements >**

**< Comment 1 >**

Please ensure that your manuscript meets PLOS ONE's style requirements, including those for file naming. The PLOS ONE style templates can be found at

<https://journals.plos.org/plosone/s/file?id=wjVg/PLOSOne_formatting_sample_main_body.pdf>

<https://journals.plos.org/plosone/s/file?id=ba62/PLOSOne_formatting_sample_title_authors_affiliations.pdf>

**< Response >**

We confirm that our manuscript meets PLOS ONE’s style requirements, including those for file naming.

**< Comment 2 >**

We suggest you thoroughly copyedit your manuscript for language usage, spelling, and grammar. If you do not know anyone who can help you do this, you may wish to consider employing a professional scientific editing service.

The American Journal Experts (AJE) (https://www.aje.com/) is one such service that has extensive experience helping authors meet PLOS guidelines and can provide language editing, translation, manuscript formatting, and figure formatting to ensure your manuscript meets our submission guidelines. Please note that having the manuscript copyedited by AJE or any other editing services does not guarantee selection for peer review or acceptance for publication.

Upon resubmission, please provide the following:

The name of the colleague or the details of the professional service that edited your manuscript

A copy of your manuscript showing your changes by either highlighting them or using track changes (uploaded as a *supporting information* file)

A clean copy of the edited manuscript (uploaded as the new *manuscript* file)”

**< Response >**

Prof. Dr. Shehzad Khalid proofread the manuscript. Both clean copy and track changes are being submitted.

**< Comment 3 >**

Please note that PLOS ONE has specific guidelines on code sharing for submissions in which author-generated code underpins the findings in the manuscript. In these cases, all author-generated code must be made available without restrictions upon publication of the work. Please review our guidelines at https://journals.plos.org/plosone/s/materials-and-software-sharing#loc-sharing-code and ensure that your code is shared in a way that follows best practice and facilitates reproducibility and reuse.

**< Response >**

The authors agree to share the code to reproduce the results of this research.

**< Comment 4 >**

We note that the grant information you provided in the ‘Funding Information’ and ‘Financial Disclosure’ sections do not match.

When you resubmit, please ensure that you provide the correct grant numbers for the awards you received for your study in the ‘Funding Information’ section.

**< Response >**

Corrected: “This work was supported by KUCARS, Department of Mechanical and Nuclear Engineering, Khalifa University under Award numbers FSU-2021-019 and RC1-2018-KUCARS.”

**< Comment 5 >**

Thank you for stating the following financial disclosure:

"This work was supported by KUCARS, Department of Mechanical and Nuclear Engineering, Khalifa University under Award numbers FSU-2021-019 and RC1-2018-KUCARS."

Please state what role the funders took in the study. If the funders had no role, please state: ""The funders had no role in study design, data collection and analysis, decision to publish, or preparation of the manuscript.""

If this statement is not correct you must amend it as needed.

Please include this amended Role of Funder statement in your cover letter; we will change the online submission form on your behalf.

**< Response >**

We amended the role of funder statement and also included in cover letter in yellow highlighted text. “Role of Funders: The funders oversaw this research and helped in preparing the manuscript.”

**< Comment 6 >**

Thank you for stating the following in the Acknowledgments Section of your manuscript:

"This work was supported by KUCARS, Department of Mechanical and Nuclear Engineering, Khalifa University under Award numbers FSU-2021-019 and RC1-2018-KUCARS."

We note that you have provided funding information that is not currently declared in your Funding Statement. However, funding information should not appear in the Acknowledgments section or other areas of your manuscript. We will only publish funding information present in the Funding Statement section of the online submission form.

Please remove any funding-related text from the manuscript and let us know how you would like to update your Funding Statement. Currently, your Funding Statement reads as follows:

"The author(s) received no specific funding for this work"

Please include your amended statements within your cover letter; we will change the online submission form on your behalf.

**< Response >**

We have renamed the “Funding and Acknowledgement” section to “Funding Statement” in the revised manuscript and updated the funding statement as follows:

**Previous statement:**

The author(s) received no specific funding for this work.

**Revised statement:**

This work was supported by KUCARS, Department of Mechanical and Nuclear Engineering, Khalifa University under Award numbers FSU-2021-019 and RC1-2018-KUCARS.

**< Comment 7 >**

We note that your Data Availability Statement is currently as follows: All relevant data are within the manuscript and its Supporting Information files.

Please confirm at this time whether or not your submission contains all raw data required to replicate the results of your study. Authors must share the “minimal data set” for their submission. PLOS defines the minimal data set to consist of the data required to replicate all study findings reported in the article, as well as related metadata and methods (https://journals.plos.org/plosone/s/data-availability#loc-minimal-data-set-definition).

For example, authors should submit the following data:

- The values behind the means, standard deviations and other measures reported;

- The values used to build graphs;

- The points extracted from images for analysis.

Authors do not need to submit their entire data set if only a portion of the data was used in the reported study.

If your submission does not contain these data, please either upload them as Supporting Information files or deposit them to a stable, public repository and provide us with the relevant URLs, DOIs, or accession numbers. For a list of recommended repositories, please see https://journals.plos.org/plosone/s/recommended-repositories.

If there are ethical or legal restrictions on sharing a de-identified data set, please explain them in detail (e.g., data contain potentially sensitive information, data are owned by a third-party organization, etc.) and who has imposed them (e.g., an ethics committee). Please also provide contact information for a data access committee, ethics committee, or other institutional body to which data requests may be sent. If data are owned by a third party, please indicate how others may request data access.

**< Response >**

In this study, we used publicly available open datasets. These publicly available datasets links are provided below:

**Data source links:**

<https://www.kaggle.com/datasets/seroshkarim/cotton-leaf-disease-dataset>

<https://data.mendeley.com/datasets/6hm6pc3y43/2>

<https://universe.roboflow.com/national-college-of-ireland/cotton-plant-disease-prediction-igthk/dataset/3>

<https://universe.roboflow.com/roboflow-100/cotton-plant-disease/dataset/2>

There are no such graphs that used mean, standard deviation, etc., values to plot. However, to reproduce the research work authors have included the details on the data augmentation (Table 3), model details (Table 4, 5, and 8).

**< Comment 8 >**

PLOS requires an ORCID iD for the corresponding author in Editorial Manager on papers submitted after December 6th, 2016. Please ensure that you have an ORCID iD and that it is validated in Editorial Manager. To do this, go to ‘Update my Information’ (in the upper left-hand corner of the main menu), and click on the Fetch/Validate link next to the ORCID field. This will take you to the ORCID site and allow you to create a new iD or authenticate a pre-existing iD in Editorial Manager. Please see the following video for instructions on linking an ORCID iD to your Editorial Manager account: https://www.youtube.com/watch?v=_xcclfuvtxQ

**< Response >**

Thanks for your update. The corresponding author has linked his ORCID with the editorial manager.

<https://orcid.org/0000-0001-7679-081X>

**< Comment 9 >**

Please ensure that you refer to Figure 12 in your text as, if accepted, production will need this reference to link the reader to the figure.

**< Response >**

Figure 12 has been referenced in the text and updated in the revised manuscript. Please see 2nd paragraph of “Results” section.

**< Comment 10 >**

We note you have included a table to which you do not refer in the text of your manuscript. Please ensure that you refer to Table 5 in your text; if accepted, production will need this reference to link the reader to the Table.

**< Response >**

Authors would like to thank the editor and have referenced the Table 5 in the text.

Please see the “Proposed Methodology” section, the paragraph starting with the following statement:

Table 5 provides detail of parameters of MobileNet V2.

**< Reviewer 1 >**

**< General Comment >**

Authors of this manuscript addressed the problem of disease detection in cotton crop and provide the solution to detect the disease by using deep learning method. This is good piece of work but it require following corrections.

**< Response >**

Thanks for your positive response and valuable comments. We have thoroughly revised our paper according to the comments. All the revisions are highlighted in red and blue text in the revised manuscript.

**< Comment 1 >**

The overall quality of the paper in general, and the implementation and presentation aspect need much improvement.

**< Response >**

We have thoroughly revised our paper by addressing the implementation and result presentation to improve the overall quality of the paper.

**< Comment 2 >**

The novelty of this research is not well explained

**< Response >**

We apologize for the confusion. We have thoroughly revised the contribution and discussion sections of our paper to highlight the novelty of this research as follows:

**Novelty of the study:**

We proposed a comprehensive system designed to effectively manage and overcome these challenges, ensuring reliable and accurate performance in diverse agricultural environments. Contributions of this research to the body of knowledge include:

- Developed a deep ensemble method to accurately classify various cotton diseases, addressing challenges of class imbalance and symptom variation through a new data augmentation scheme and feature fusion approach.
- Proposed a synthetic data augmentation scheme using a customized StyleGAN along with conventional methods (scaling, rotating, transforming, shearing, zooming) to tackle class imbalance.
- Introduced a dual-stage fusion scheme by combining features extracted from MobileNet and VGG16 to create a comprehensive feature vector, followed by a StackNet-based ensemble classifier integrating Long and Short Term Memory (LSTM), Support Vector Machine (SVM), and Random Forest for enhanced prediction accuracy.
- Achieved an average accuracy of 97% on a publicly available dataset, surpassing state-of-the-art techniques in identifying six diseases and a healthy class.

These modifications are included at the end of the “Introduction” section.

**Key Findings and Potential Significance:**

The proposed ensemble method, trained with categorical cross-entropy loss, RMSProp optimizer, and softmax activation, achieves a 97\% accuracy. This research introduces several novel aspects of precision agriculture, particularly in detecting and managing crop diseases and developing a robust method that deals with real-world challenges such as varying disease symptoms, occlusion from overlapping leaves, and diverse leaf characteristics in shape, size, and texture. A novel data augmentation technique has been developed to address the issue of data scarcity and class imbalance in agricultural datasets. Incorporating multi-convolutional neural networks combined with ensemble classification techniques is a novel approach to improve the accuracy of disease detection. This method combines the strengths of convolutional neural networks for feature extraction and classification. As a result, it accurately identifies six specific diseases and a healthy class in cotton crops.

The above paragraph highlighting the novelty of this study is also added as the last paragraph in the “Discussion” section.

**< Comment 3 >**

“A robust system capable of addressing real-life challenges, such as diverse symptoms of diseases, occlusion, and variations in leaves” what is meaning by real life challenges is not clear

**< Response >**

We apologize for the vague explanation. We revised these lines as follows:

A robust deep learning system is essential for addressing the practical challenges encountered in real-world agricultural settings. These challenges include diverse symptoms of diseases, such as varying sizes and colors of lesions, which can complicate accurate diagnosis. Additionally, occlusion, where leaves are partially covered by other leaves or objects, poses a significant obstacle to clear image capture and analysis. Variations in leaf appearance, including differences in shapes, sizes, and textures, further add to the complexity. To tackle these issues, we proposed a comprehensive system designed to effectively manage and overcome these challenges, ensuring reliable and accurate performance in diverse agricultural environments.

These lines are updated at the end of the “Introduction” section (before the 1st bullet point of the contribution part).

**< Comment 4 >**

Classification could be done by using SVM and random forest but LSTM is used for sequential data why all these are used collectively is not clearly explained.

**< Response >**

Thanks for your valuable comment. We added further details to the proposed methodology to explain the significance of LSTM, which is used along with SVM and RF as followas:

The use of LSTM, despite its typical application for sequential data, is beneficial as it captures temporal dependencies or patterns within the feature vectors that traditional classifiers like SVM and Random Forest might overlook. By integrating these diverse methods, the strength of LSTM in handling sequential patterns is leveraged alongside the robustness of Random Forest in managing non-linear relationships and the effectiveness of SVM in high-dimensional spaces, thereby enhancing overall model performance.

Please see the 2^nd^ paragraph of the section “Proposed Methodology”.

**< Comment 5 >**

How the stylGANs approach is addressing the problem of Data scarcity is not clear.

**< Response >**

Explanation is added to clarify how styleGANs help with data scarcity as follows:

StyleGANs address the problem of data scarcity by generating high-quality synthetic images that are indistinguishable from real images. This increases the dataset size and ensures diversity and variety in the augmented data, thus enhancing the model's ability to generalize better.

Please see the 3^rd^ paragraph of the section “Proposed Methodology”.

**< Comment 6 >**

Authors claimed that their method is outperforming than the existing methods but the comparison is not clear

**< Response >**

We apologize for the vague explanation. We have thoroughly revised the comparison table and highlighted the performance gains of the proposed method compared to existing methods as follows:

**Comparison**

Table 8 presents a comparative analysis of the proposed model versus existing methods for cotton disease detection, focusing on their parameters (#Par in millions) and accuracy. The proposed ensemble model achieves the highest accuracy of 0.97, indicating a 6% performance gain over the next best models. The second-best models [50, 54], with accuracies of 0.91 and 0.90, include MobileNetV2 with a learning scheduler (0.91, 3.5M), MobileNetV2 with more dense layers (0.90, 3.5M), and EfficientNet (0.90, 5.3M). The third-best models [48, 50, 51], with an accuracy of 0.88, include ResNet152V2 (60.2M), MobileNetV2 (3.5M), MobileNetV2 unfreezing initial layers (3.5M), Xception (22.9M), and ResNet152V2 with a learning rate scheduler (60.2M). Despite the proposed method's high parameter count (143.4M), its 6% performance gain justifies the complexity, significantly outperforming models like MobileNetV2 (3.5M) and DenseNet121 (8.0M), which achieve relatively high accuracy. This highlights the effectiveness of our ensemble model in achieving superior accuracy, balancing parameter size and performance gain, and demonstrating the model's efficacy in cotton disease detection applications.

Our newly added “Comparison” subsection includes these lines under the “Results” section.

**< Reviewer 2 >**

Dear Reviewer,

Thanks for your positive response and valuable comments. We have thoroughly revised our paper according to the comments. All the revisions are highlighted in red and blue text in the revised manuscript.

**< Comment 1 >**

The author implement state of art method like UNet or UNet++ for segmentation. Fig. 4 should be application specific relevant to the proposed work...it looks as general figure.

**< Response >**

Thanks for your valuable comment. We revised this figure to make it specific to the leaf disease as follows:

**Previous figure:**

**Revised figure:**

We updated the revised figure in the final version of our paper.

**< Comment 2 >**

Table 7 is not clear, the values represent what?

**< Response >**

We revised this Table and add the additional explanation as follows:

Table 7 presents the accuracy ratings of various classifiers using feature sets extracted from VGG16 and MobileNetV2 deep learning models, including Decision Tree (DT), k Nearest Neighbor (KNN), Long short-term memory units (LSTM), Random Forest (RF), Support Vector Machine (SVM), and an Ensemble Classifier (StackNet). The Decision Tree classifier shows a decrease in performance with MobileNetV2 features (62% to 56%), while KNN demonstrates a significant improvement (65% to 87%). The LSTM classifier performs well with both feature sets, showing a slight improvement from 84% to 87%, and the Random Forest classifier also improves moderately from 80% to 83%. The SVM classifier maintains high accuracy, improving slightly from 86% to 88%. Notably, the Ensemble Classifier (StackNet) outperforms all individual classifiers by a significant margin, achieving a consistently high accuracy of 97% with both feature sets. The percentage gain of the Ensemble Classifier over the best individual classifier (SVM) is approximately 12.79% for VGG16 features and 10.23% for MobileNetV2 features, highlighting the robustness and effectiveness of the proposed method in integrating multiple model predictions.

Please see the “Ablation Study” subsection of “Proposed Methodology” section.

**< Comment 3 >**

Discuss the related work in such a manner that it should highlight theme, results, advantages, and limitations. Add 8-10 more literature and discuss it properly. The final review analysis should be added at the end of this section. It will improve the readability of the paper. Add papers from 2021-2023, also Discussion section should contain the result impact, comparative study analysis and the overall analysis of the result.

**< Response >**

More papers are added from 2021-2024 and literature review is expanded according to the instructions. Discussion section is also updated with the result impact and comparative study analysis and the overall result analysis.

Table 2 is added under the section of “Related Work”

**< Comment 4 >**

limitations and future scope need to be included.

**< Response >**

Thank you for your valuable feedback. We included a separate subsection “Limitations and Future Directions” to highlight the limitations and future work of this study as follows:

**Limitations and Future Directions:**

The primary limitation of this study is the possibility of overfitting due to the controlled environment in which the data was collected and the models were trained. This controlled setting may not accurately reflect the variability and complexity of real-world field conditions, leading to lower performance when the models are applied outside of the controlled environment. Additionally, the reliance on a relatively small dataset restricts the system's effectiveness and its ability to generalize well across diverse scenarios.

Future research should focus on real-time data acquisition and processing to enhance model robustness across various real environments, mitigating overfitting risks. Improving model interpretability and transparency will ensure AI decisions are understandable to farmers and agricultural professionals. Developing user-friendly interfaces and mobile applications will aid practical field use. Expanding the dataset with diverse images of diseased leaves will improve generalization and accuracy. Advanced algorithms for precise disease coverage localization will optimize pesticide usage, while refined segmentation techniques will better handle overlapping symptoms of multiple diseases on a single leaf.

Please check the subsection “Limitations and Future Directions” of the “Discussion” section.
